# Transcriptomic Analysis of the Interaction Between *FLOWERING LOCUS T* Induction and Photoperiodic Signaling in Response to Spaceflight

**DOI:** 10.3389/fcell.2021.813246

**Published:** 2022-02-01

**Authors:** Lihua Wang, Junyan Xie, Chenghong Mou, Yuwei Jiao, Yanhui Dou, Huiqiong Zheng

**Affiliations:** ^1^ Center for Excellence in Molecular Plant Sciences, Chinese Academy of Sciences, Shanghai, China; ^2^ University of Chinese Academy of Sciences, Beijing, China

**Keywords:** photoperiod, spaceflight, transcriptome, *Arabidopsis thaliana*, reproductive stage

## Abstract

Spaceflight has an impact on the growth and development of higher plants at both the vegetative stage and reproductive stage. A great deal of information has been available on the vegetative stage in space, but relatively little is known about the influence of spaceflight on plants at the reproductive stage. In this study, we constructed transgenic *Arabidopsis thaliana* plants expressing the flowering control gene, *FLOWERING LOCUS T* (*FT*), together with the green fluorescent protein gene (*GFP*) under control of a heat shock-inducible promoter (*HSP17.4*), by which we induced *FT* expression inflight through remote controlling heat shock (HS) treatment. Inflight photography data showed that induction of *FT* expression in transgenic plants in space under non-inductive short-day conditions could promote flowering and reduce the length of the inflorescence stem in comparison with that of wild-type plants under the same conditions. Whole-genome microarray analysis of gene expression changes in leaves of wild-type and these transgenic plants grown under the long-day and short-day photoperiod conditions in space indicated that the function of the photoperiod-related spaceflight responsive genes is mainly involved in protein synthesis and post-translation protein modulation, notably protein phosphorylation. In addition, changes of the circadian component of gene expression in response to spaceflight under different photoperiods indicated that roles of the circadian oscillator could act as integrators of spaceflight response and photoperiodic signals in Arabidopsis plants grown in space.

## Introduction

Spaceflight conditions, including microgravity, could cause an impact on growth and development of higher plants at both the vegetative stage and the reproductive stage. A great deal of information is available on the vegetative stage in space. For example, alteration of auxin polar transport in etiolated pea seedlings and maize coleoptiles in space (Ueda et al., 2000), inhibition of cell division and mitosis, as well as significant karyological disturbances in the root-tip cells of oat, mung bean, and sunflower seedlings grown in space, and the modification of cell wall metabolism (Krikorian and O’Connor, 1984; Rasmussen et al., 1994; Soga et al., 2002). Reduction in the fresh weight of shoots and photosynthetic function of wheat plants grown onboard a space shuttle (Tripathy et al., 1996). Most plant seedlings grown in space were smaller than comparably aged ground controls (Kiss et al., 2000; Paul et al., 2012; Wang et al., 2018), while some seedlings grew faster in space (Matía et al., 2010; Hoson et al., 2014). However, relatively little is known about the influence of spaceflight on plants at the reproductive stage, including flowering. Some early experiments reported failure in seed formation under spaceflight conditions (Kuang et al., 1996; Strickland et al., 1997; Soga et al., 1999; Levinskikh et al., 2000; Campbell et al., 2001). As hardware improved, the seed-to-seed cycle of several plants in space was achieved (Link et al., 2003; Sychev et al., 2003; Link et al., 2014). These results indicated that plants could adapt to spaceflight for seed-to-seed growth, but reproductive fitness was often reduced in space (De Micco, et al., 2014). Interruption of the reproductive process, delay in the completion of a single reproductive phase, the lowering of reproductive success and alteration of seed reserves are still major bottlenecks to maximizing the efficiency of plant growth and reproduction in space, as well as to be used to support life in long-term manned missions (Hoson et al., 2014; reviewed by; Zheng 2018).

The reproductive success of plants is often dependent on their flowering time being adapted to the growth environment. Several studies suggest that both biotic and abiotic stress factors play key roles in controlling the alteration of flowering time and architecture for reproductive fitness in plants. For example, plants often accelerate the flowering process under drought stress (Sherrard and Maherali, 2006; Galbiati et al., 2016) and delay flowering time by salt stress (Achard et al., 2006; Ma et al., 2015). Heat and cold stress can also have a dramatic effect on flowering and impaired concomitant change of architecture for continued resource production through photosynthesis. Increasing evidence documents that spaceflight conditions are novel stressors for plants grown in space, which causes changes at the physiological, morphological, and molecular levels, including altered transcription patterns of many genes (Paul et al., 2001; De Micco et al., 2014; Zhang et al., 2015; Xie and Zheng, 2020; Karahara et al., 2020). In the space-grown Mizunna, a total of 20 in 32 ROS oxidative maker genes were up-regulated, including the common genes’ responses to abiotic and biotic stress (Sugimoto et al., 2014). In Arabidopsis culture cells grown in space, genes associated with heat shock, salt, drought, metals, wounding, phosphate, ethylene, senescence, terpenoids, seed development, cell walls, photosynthesis, and auxin were up-regulated by five folds in comparison with their ground controls (Paul et al., 2012; Kwon et al., 2015). The endogenous systems that measure day length were found to interact with stress responses and override interpretation of the signals in plants on the ground (Becker et al., 2005). It is however unclear how the photoperiod influences the reproductive fitness of plants during spaceflight.

The developmental rate of Arabidopsis plants on the ground is directly related to daylength, because it is a long-day (LD) plant, an increase in photoperiod results in an increase in development rate. How photoperiod affects plant development in space is yet to be known. No space experiments had been carried out to compare the effects of different photoperiods on plant growth and development so far. To examine the effects of photoperiod signals on the spaceflight response of plants during the reproductive stage, we conducted the space experiment by growing Arabidopsis plants under the LD and the short-day (SD), respectively, on board the Chinese recoverable satellite SJ-10. A transgenic plant expressing *FLOWERING LOCUS T* (*FT*) and the reporter gene green fluorescent protein (GFP) under control of a heat shock-inducible promoter (*HSP17.4*) was constructed to investigate the role of *FT* expression in plants at the reproductive stage in space in counteracting the stresses of spaceflight and regulation of the reproductive development. In addition, a full-genome analysis of RNA derived from the leaves of Arabidopsis plants grown under the LD and the SD during spaceflight, respectively, was also performed in comparison with their controls on the ground. As the plants for the transcription analysis in this study were sampled about 2 h post-landing, we use “spaceflight” (not microgravity) to include altered gravity during landing and other spaceflight environmental factors.

## Materials and Methods

### Plant Materials and Growth Conditions

*Arabidopsis thaliana* Columbia (Col-0) ecotype was used as the wild-type (WT). Plants were germinated and grown in plastic cups under long-day (16 h light/8 h dark, LD) at 120 μmol m^−2^. s^−1^ conditions for 5 days, then set in the root modules (240 × 120 × 65 mm^3^) containing a commercially available vermiculite immersed by a medium containing MS macronutrients (Murashige and Skoog, 1962) and cultured in a greenhouse under the LD or the short-day (16 h dark/8 h light, SD) for an additional 15 days prior to flight.

### Construction of Transgenic Arabidopsis Plants

For the construction of *pHSP*:*FT*, the coding sequence (CDS) of *FT* was amplified by PCR from a WT (Col-0) cDNA using the primers with the restriction sites underlined 5′-ATCACT​AGTATG​TCT​ATA​AAT​ATA​AGA​GAC​CCT​CTT​A-3′ and 5′- CGTTCT​AGACTA​AAG​TCT​TCT​TCC​TCC​GCA​GC-3′ and ligated into a pBluescript KS minus vector. A 1109 bp DNA fragment, upstream from *HSP17.4* (AT3G46230) start codon corresponding to the putative promoter, was amplified by PCR with the primers 5′-ACTCTG​CAGACC​AGT​CAT​ACG​TAG​CGC​AAT-3′ and 5′-ATGACT​AGTCGT​TTC​GCT​TAC​TCT​GTT​TGC-3′, and fused to the *FT* coding sequence in the pBluescript KS minus vector. For the construction of *pHSP*: *GFP*, the *HSP17.4* promoter DNA fragment was fused to a *GFP* in pBluescript SK minus vector. *pHSP:FT* or *pHSP:GFP* were then cleaved and ligated into the *p*Cambia1301-NOS-3^**,**^ vector. These two gene fusions were transferred to Arabidopsis (Col-0) plants through the *Agrobacterium tumefaciens* strain GV3101, respectively, by the floral dip method according to the methods of Clough and Bent (1998). After regeneration in the presence of hygromycin, transgenic plants were screened for expression of *FT* and *GFP* by heat shock (37°C for 1 h) in an incubator. *pHSP*:*FT*, *pHSP*:*GFP* (FG) gene were co-expression in Arabidopsis by genetic crossing as described by Qi and Zheng (2013). F3 progeny homozygous for FG were used for space experiments.

### Hardware Design and the Spaceflight Experiment

**FIGURE 1:**
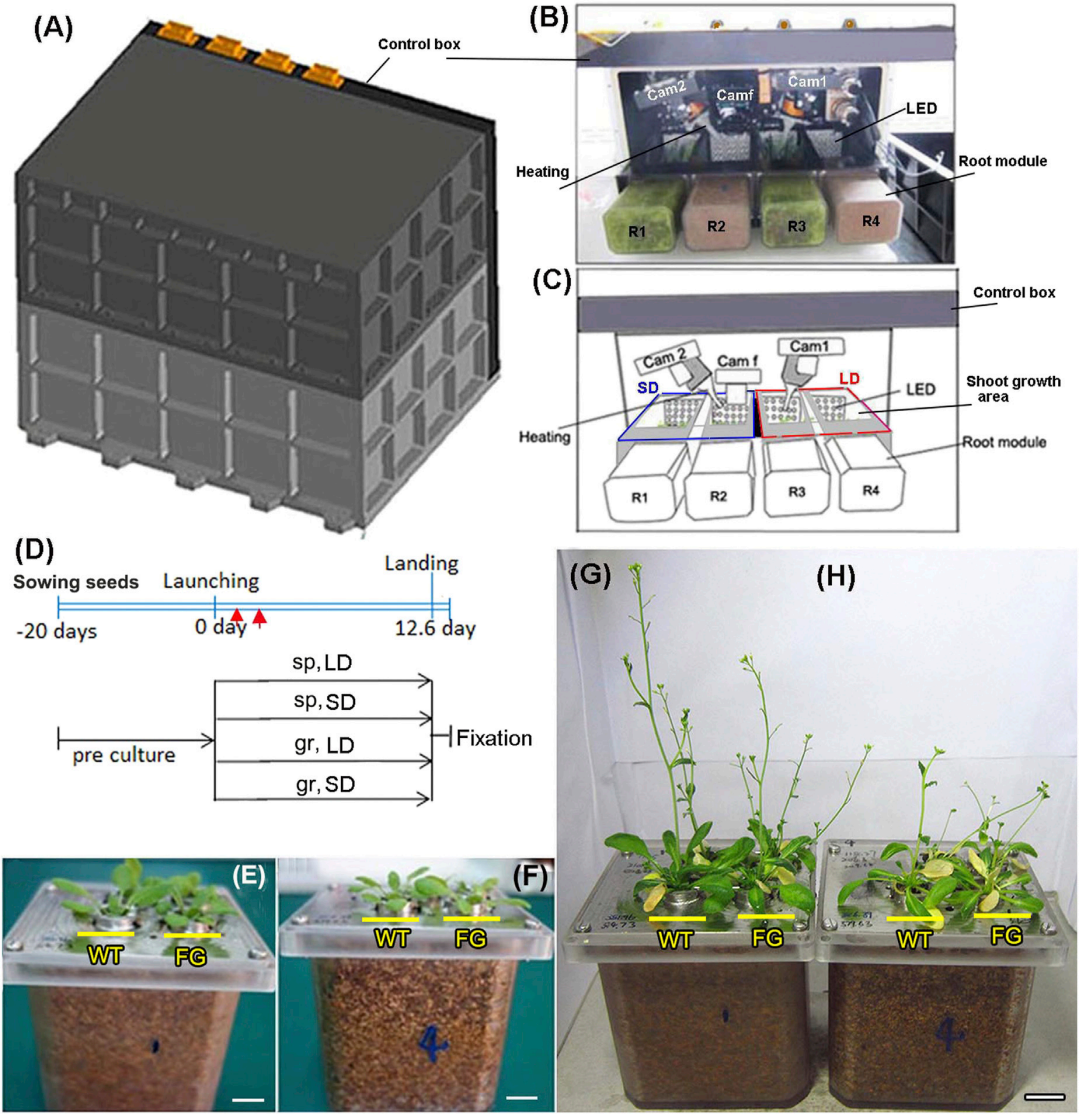
Experimental setup on board the Chinese recoverable satellite SJ-10. **(A)** The external view of the plant growth unit (PGU) (H×W×D = 370 × 270 × 270 mm) with the cover. **(B)** The inside view of the PGU without the cover, as described in **(C)**. **(C)** Diagram of PGU used on board the Chinese satellite SJ-10. Showing the distribution and state of components and samples in PGU during space flight. The components included four root modules, three cameras, two fans and heating parts, and four LED banks with controlled a long-day (LD, 16 h light/8 h dark) and a short-day (SD, 8 h light/16 h dark) photoperiods, respectively. LED plates, heating systems, video CCD cameras (Cam 1 and Cam 2), the GFP imaging camera (Cam f) and root modules (R1 and R3 with rice seedlings; R2 and R4 with Arabidopsis seedlings) are indicated. **(D)** An overview of the time process of the space experiment. wild-type (WT) and transgenic (*pHSP:FT; pHSP:GFP, FG*) plants were germinated and grown in the root module in green house on ground for 20 days after sowing (pre-culture). At this age, all plants had six to seven rosette leaves were selected to load into the PGU less than 24 h prior to took off. Heating treatment (red arrows point) to activate expression of *FT* and *GFP* was performed at day 2 on orbiter. The plants in the PGU were grown under the LD and the SD conditions in space (sp) and on ground (gr), respectively. **(E,F)** 20-day old WT and FG plants grown in the root modules before being loaded into chambers of the PGU under the LD **(E)** and the SD **(F)** conditions, respectively. **(G,H)** The situation of samples of **(E)** and **(F)** recovered from spaceflight under the LD **(G)** and the SD **(H)** conditions in the PGU. Bars = 1 cm.

The plant growth system used for the SJ-10 experiment consisted of four growth compartments, illumination, photograph, air-flowing heating, and humidity controlling system (Figures 1A–C). Plants had about six to seven rosette leaves when they were loaded into the growth chamber (Figures 1E,F). Two set of plants were prepared and placed in the plant growth units (PGUs) for spaceflight experiment (Figures 1A–C) and the ground control, respectively. The flight PGU was positioned in the capsule of satellite SJ-10 about 8 h prior to launch. The SJ-10 satellite was in orbit for about 12 days and 15 h (launch: 1:38, April 6, 2016; landing: 16:30, April 18, 2016). Illumination was provided by light banks made up of 200 solid state light emitting diode (LED) lamps (400–700 nm white light and red light, 2:1) in LD (16 h light/8 h dark) and SD (8 h light/16 h dark) photoperiod conditions, respectively. Inside the chambers, temperatures were 22 ± 2°C, relative humidity was between 90 and 100%. The photosynthetically active photon flux density produced by LED lamps was 120 μmol m^−2^. s^−1^ for Arabidopsis at the surface of the first leaf of the experimental plants. Temperature and humidity were recorded every 1 min during flight. These data were used to set the ground control in a control PGU. Three digital cameras were mounted in the PGU to allow the recording of plant growth and development of plants in space. Photographic equipment consisted of two digital cameras (image size 1,280 × 1,024 pixels) and one GFP fluorescence camera, which was automatic and preprogrammed and allowed the recording of plants in PGUs both in visible light and in GFP fluorescence (Figures 1B,C). The photographs were taken at 2 h intervals during the light period. Two digital cameras were used for photographed seedlings grown under the LD and the SD conditions, respectively. The GFP fluorescence camera was used to follow the expression of GFP in seedlings after heating was induced (Figure 1D). All manipulations involved in the experiment were automated or carried out by remote control.

After SJ-10 satellite return to Earth, the PGU was unloaded and received at a temporary laboratory in the landing site about 2 h post-landing (Figures 1G,H). Plants were harvested and fixed with RNAlater solution (ambion, Austin, TX, United States ) at the landing site. The samples were then brought to our Shanghai laboratory where they were analyzed for transcriptional changes.

### Sample Processing and Analysis

Total RNA was extracted from rosette leaves of WT plants and FG plants grown under the LD and the SD in space and on ground, respectively. Each treatment included four full expansion rosette leaves from two different plants. The RNA was then purified using miRNeasy Mini Kit (Cat#217004, QIAGEN, GmBH, Germany) following the manufacturer’s instructions and checked for a RIN number to inspect RNA integration by an Agilent Bioanalyzer 2,100 (Agilent technologies, Santa Clara, CA, US). RNA was amplified, labeled, and purified by using GeneChip 3′ IVT PLUS Reagent Kit (Cat#902416, Affymetrix, Santa Clara, CA, US) following the manufacturer’s instructions to obtain biotin labeled cRNA. Array hybridization and wash was performed using GeneChip® Hybridization, Wash and Stain Kit (Cat#900720, Affymetrix, Santa Clara, CA, US) in Hybridization Oven 645 (Cat#00–0331-220V, Affymetrix, Santa Clara, CA, US), and Fluidics Station 450 (Cat#00–0,079, Affymetrix, Santa Clara, CA, US) following the manufacturer’s instructions.

### Analysis of Microarray Data

Slides were scanned by GeneChip® Scanner 3,000 (Cat#00–00212, Affymetrix, Santa Clara, CA, US) and Command Console Software 4.0 (Affymetrix, Santa Clara, CA, US) with default settings. Raw data were normalized by MAS 5.0 algorithm, Affymetrix packages in R. Probe sets with signal values lower than the detectable range were adjusted to 75 and probe sets with the values of 75 for all conditions were removed from subsequent analysis. The averages of normalized ratios were calculated by dividing the average of the normalized signal channel intensity by the average of the normalized control channel intensity. The standard deviation of the ground control (two biological replicates) was employed to identify genes of significant changes relative to the ground controls (*p* value < 0.05). Only genes that showed transcript level changes in at least two folds in comparison with its ground control and with the same tendency in both biological replicates were considered as relevant for spaceflight. Gene Ontology (GO) over representation was performed using PANTHER (Fisher’s exact type with false discovery rate correction, http://www.pantherdb.org, Mi et al., 2019). For motif enrichment, motifscan (Sun et al., 2018) was used to determine whether the occurrence of a given motif in input genes was significantly high as compared with that in random regions (Ran et al., 2019).

### Quantitative Real-Time RT-PCR

Total RNA was extracted from leaves of the space samples and the ground controls as described. The genes and their qRT-PCR primers are presented in Supplementary Table S1. The Arabidopsis *ACTIN* gene (At3g18780) was used as a quantitative control for all qRT-PCRs. At least three technical replicates of each biological replicate were used for real-time PCR analysis.

## Results

### Gene Switch for Inducing Expression of *FT* in Arabidopsis Plants During Spaceflight

To address the effects of spaceflight on the *FT* regulating reproductive development, we generated transgenic Arabidopsis plants that stably harbor *FT* and *GFP*, under the control of the *HSP17.4* promoter (*pHSP*:*FT, pHSP*:*GFP*). Under the LD conditions, these *pHSP*:*FT, pHSP*:*GFP* (FG) transgenic plants grown on the ground at normal temperatures (22 ± 2°C) exhibited a phenotype like WT, except the size was slightly smaller and flowering took place early (Supplementary Figures S1,S2). In the absence of heat shock (HS), no GFP fluorescence in leaves of FG plants was observed (Supplementary Figure S1C), while heating at 37°C for 1 h resulted in a clear induction of GFP expression in the leaves of FG plants (Supplementary Figure S1D), but did not in the leaves of WT plants (Supplementary Figure S1B). Early floral development and apparent increase of *FT* gene expression in FG plants under the SD conditions (Supplementary Figure S2) were also observed after HS induction, while the control plants (WT and *pHSP*:*GFP*) with or without exposure to HS treatments showed negligible levels of background *FT* expression and little GFP fluorescent under the same conditions (Supplementary Figures S1,S2).

**FIGURE 2:**
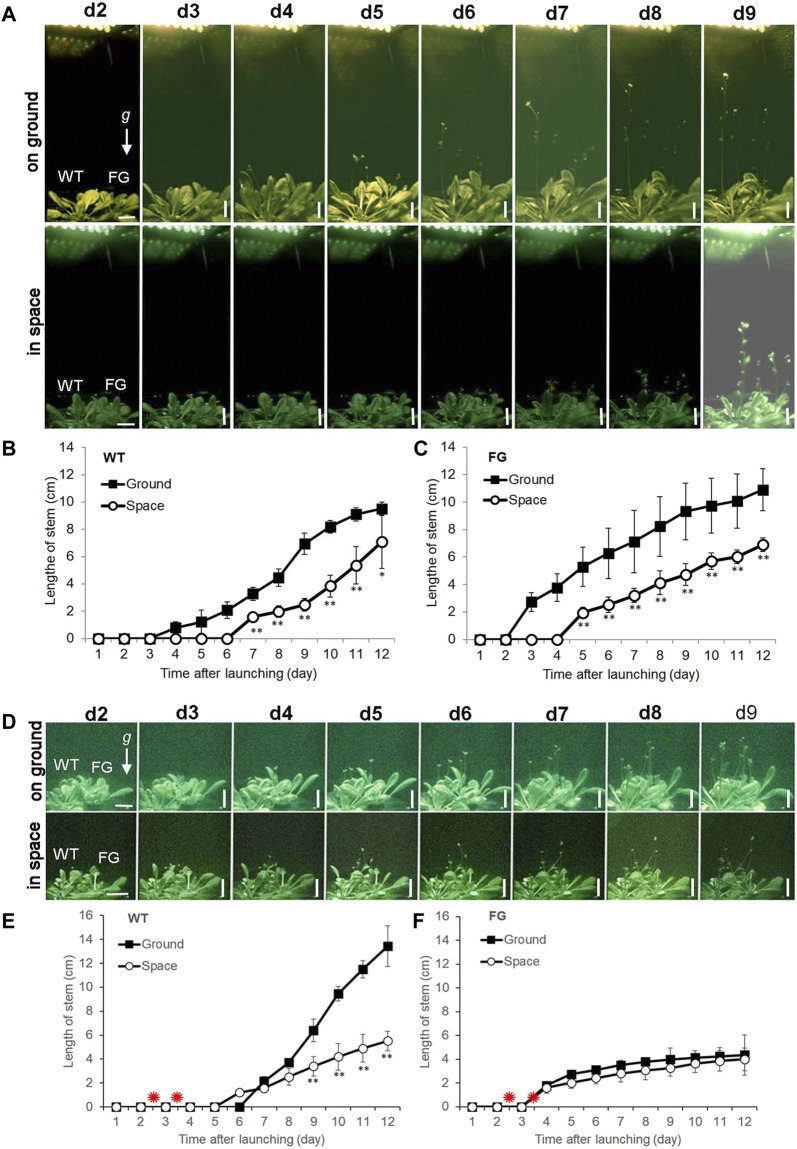
Floral transition time of wild-type (WT) and *pHSP*:*FT*; *pHSP*:*GFP* (FG) transgenic plants grown under the long-day (LD) and the short-day (SD) conditions on the ground and in space on board the SJ-10 satellite. **(A)** Example images of plants grown under the LD condition on ground and in space at day 2 (d2) to day 9 (d9) after the SJ-10 took off. Scale bars = 10 mm. **(B,C)** Comparison of floral transition time and average length of stems of WT and FG transgenic plants under the LD in space with their controls on ground. Data are determined from the downlined images of plants on orbit in space or on ground. Error bars indicate the Standard deviation of the mean for the length of stems from the four plants (*p < 0.05, **p < 0.001, Student’s t-test). **(D)** Example images of plants of WT and FG plants under the SD condition on ground and in space at d2 to d9 after the SJ-10 took off. Scale bars = 10 mm **(E,F)** Comparison of floral transition time and average length of stems of WT and FG plants treated by heating shock (red asterisks indicate) under the SD condition in space and on ground. Data are determined from the images downlined from the PGU in space and on ground. Error bars indicate the Standard deviation of the mean for the length of stems from the four plants per treatment (**p < 0.001, Student’s t-test).

For space experiments on the satellite SJ-10, seeds of WT and FG were germinated and grown in the root modules on ground under the LD and the SD conditions, respectively, for 20 days (Figure 1E; corresponding to stage 1.06, Boyes et al., 2001). At this age, the plants had formed about six to seven rosette leaves (Figures 1E,F), when they took off. Under the LD condition, the first bolting (start of peduncle growth) of WT plants on the ground appeared at day 4 after the satellite launched, while plants in space initiated bolting on day 6 after take-off (Figures 2A,B). For FG plants, bolting appeared at day 2 under the LD on the ground, earlier than those in space at day 4 (Figures 2A,C), and slightly earlier than WT under the same condition.

**FIGURE 3:**
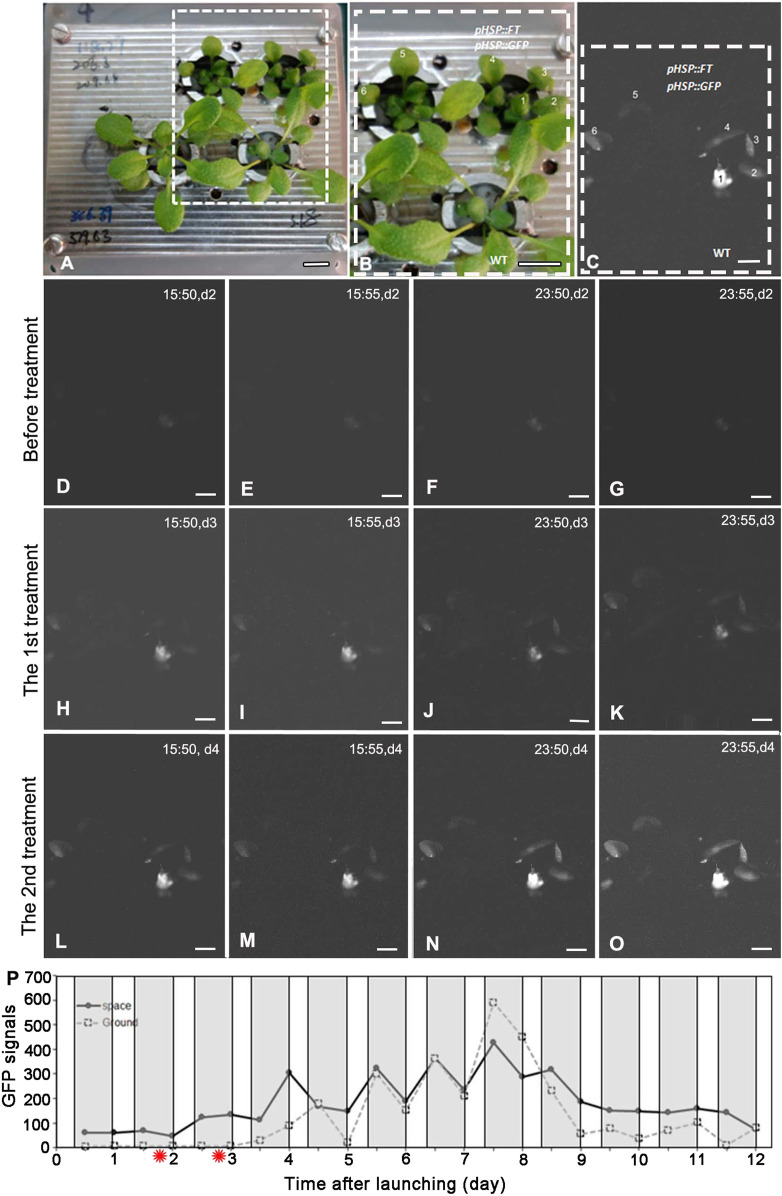
GFP expressing in plants grown under the short-day treated by heat shock on board SJ-10. **(A,B)** *pHSP*:*FT*; *pHSP*:*GFP* transgenic plants (FG) and wild-type (WT) plants photographed in white light before launching. The framed region in **(A)** is detailed in **(B)**. Bars = 10 mm. **(C)** A represent GFP image showed the expression of GFP in leaves one to six of plants in (B). Bar = 5 mm. **(D–O)** Example GFP images were captured by the GFP imager at day 2 to day 4 after heating treatment. The images were captured at four time points (15:50, 15:55, 23:50 and 23:55 on board SJ-10 satellite time) every day and reflect heating induction (37°Cfor 1 h, twice: the first treatment and the second treatment) of GFP expressing. Bars = 2.5 mm. **(P)** Quantification of the intensity of GFP signal in plants grown under the short-day (8 h light/16 h dark) in the PGU on board the SJ-10 in space and on the ground, respectively. The GFP signals was measured as described in Materials and Methods. Values represent means for two time point images (at 15:50 and 15:55 dark period and 23:50 and 23:55 light period every satellite day, respectively). The red asterisks indicate the time points of heat shock. White areas, light; dark grey panels, dark.

Under the SD condition, both WT and FG plants on the ground and in space were treated by 37°C HS for 1 h at day two and day three after take-off, respectively. No apparent GFP signal was detected in the leaves of WT and FG plants before the HS induction (Figures 3D,E). A strong transient expression of the transgenic GFP fluorescence in the leaves of FG plants in space and on the ground was detected under the SD condition at 24 h after the first HS-treatment, while no signal appeared in leaves of WT plants under the same conditions (Figure 3H–O). The highest abundance of GFP signal in the leaves of HS-treated plants grown under SD in space was observed at day 8 (Figure 3P). Flowering of the HS-treated WT plants under the SD in space showed a slight earlier flowering (about at Day 5) than those on the ground (at Day 6, Figures 2D,E), while the HS-treated FG plants under the SD showed similar flowering time (at Day 3) between in space and on the ground (Figures 2D,F). Overall, FG under SD exhibited 2∼3 days earlier flowering than that of the HS-treated WT plants under the same conditions (Figure 2D). In addition, the stem elongation of HS-treated FG plants in spaceflight under the SD was similar to the ground control (Figure 2F), while stem growth of WT plants in space under the same conditions was significantly slower than that of the ground control (Figure 2E). These results indicated that the *pHSP:FT, pHSP:GFP* system we constructed in this study could mediate an “on/off” situation of *FT* gene activity in FG plants by HS treatment and could be used as a gene switch for flowering induction both in space and on the ground.

### Identification of Differential Expressed Genes Under Different Photoperiodic Conditions in Response to Spaceflight

**FIGURE 4:**
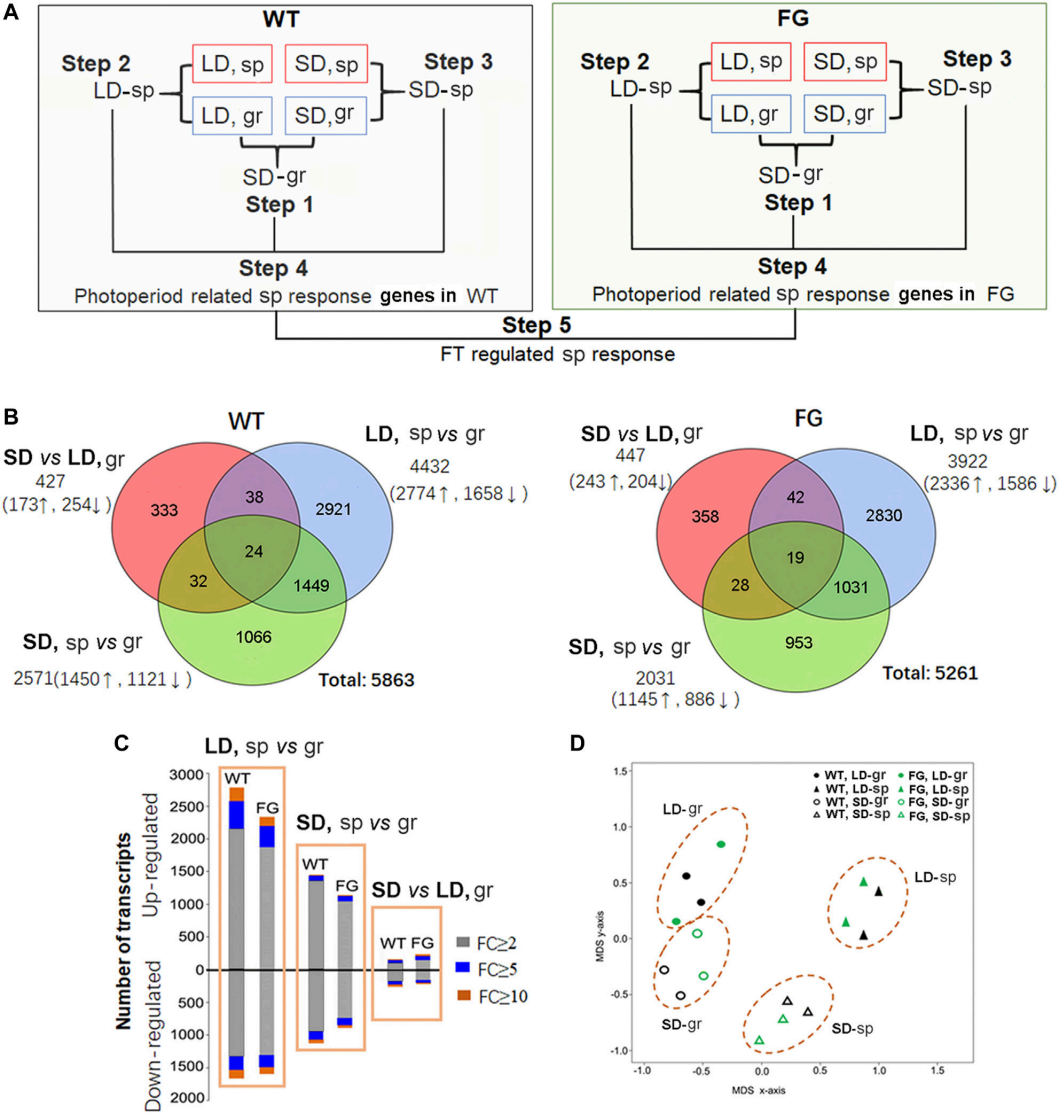
Transcriptional responses to spaceflight in wild-type (WT) and transgenic plants (*pHSP:FT*; *pHSP:GFP*, FG) under the long-day (LD) and the short-day (SD) conditions, respectively. **(A)** Workflow of microarray data analysis. Analysis of differential expression genes (DEGs) consists of five major steps: Step 1, analysis of SD photoperiod responsive genes in WT and FG plants grown on ground (gr) (SD-gr). Step 2, identification of DEGs in WT and FG plants under the LD in response to spaceflight (sp) in comparison with their controls on ground (LD-sp). Step 3, identification of DEGs in WT and FG plants under the SD in response to sp in comparison with those on gr (SD-sp). Step 4**,** Compared LD-sp DEGs in WT and FG plants with SD-sp DEGs, respectively. Step 5**,** Compared DEGs in WT with those in FG to identify the DEGs involved in FT pathway. **(B)** Venn diagram of transcriptomic data. **(C)** Numbers of DEGs in WT and FG plants (*p* < 0.05) under the LD and the SD conditions, respectively, in response to sp. FC, fold change. **(D)** Principal component analysis demonstrates a strong difference between transcriptome of the sp- and the gr-sample under the LD and the SD conditions, respectively. Multidimensional scaling (MDS) of all DEGs in WT and FG under the LD and the SD, respectively, in response to sp.

To identify the molecular basis of the influence of spaceflight conditions on plants at the reproductive stage, the global transcriptional changes were monitored in plants grown in space in comparison with their controls on the ground using whole-genome Arabidopsis GeneChips (Affymetrix). RNA was extracted from leaves of WT and FG grown in space (sp) under the LD (LD, sp) and the SD (SD, sp), and their controls on the ground (gr) under the LD (LD, gr) and the SD (SD, gr), respectively. The estimated mean level of gene expression in WT or FG in space was significantly different compared with the estimated mean of controls on the ground when controlling the false discovery rate (FDR) at the level of 0.05 using the method of storey and Tibshirani (2003). Of the genes that met these criteria, we rank ordered them by fold change (FC). Those expression levels that changed more than two-fold (FC ≥ 2) were selected as differential expression genes (DEG). The DEGs were analyzed through a series of comparisons including five steps (Figure 4A). Step 1, expression of genes in leaves grown under the SD on the ground was compared with those under the LD on the ground (SD vs LD, gr, namely SD-gr). This approach allowed us to overview the influence of the photoperiod on gene expression in WT and FG, respectively, on the ground (Supplementary Tables S2,3). Step 2, to select genes with altered expression in response to spaceflight under the LD, gene expression in WT and FG plants grown under the LD in space were compared with those under the LD on the ground (LD, sp *vs* gr, namely LD-sp), respectively (Supplementary Tables S4,5). Step 3, to identify gene expression changes in response to spaceflight occurring in WT and FG plants under the SD condition (SD, sp *vs* gr, namely SD-sp) (Supplementary Tables S6,7). Step 4, photoperiod related spaceflight responsive genes were selected by comparisons between LD-sp and SD-sp responsive genes in WT and FG plants, respectively (Supplementary Tables S8,9). Step 5, comparison of altered expression of photoperiod related spaceflight responsive genes between WT and FG was performed (Supplementary Table S10). Using this strategy, FT-regulated and photoperiod-related spaceflight responsive genes were accordingly identified.

### A Major Impact of Spaceflight on Global Transcription

Figure 4B showed that transcript abundance of 427 genes (7.3% of total 5,863 DEGs) in leaves of WT plants and 477 genes (8.5%) in leaves of FG plants grown under the SD on the ground were altered in comparison with those under the LD on the ground (Supplementary Table S2,3). Expression of 4,432 genes (75.6%) in leaves of WT and 3,922 genes (74.5%) in leaves of FG plants grown under the LD in space were altered (Supplementary Tables S4,5), while 2,571 genes (43.9%) in leaves of WT and 2031genes (38.6%) in leaves of FG grown under the SD in space changed expression levels in comparison with their controls on ground (Supplementary Tables S6,7). The proportion of genes up-regulated during spaceflight was always higher than the down-regulated ones (Figure 4C). Principal component analysis (PCA) of the samples demonstrated a strong difference between transcriptomes of samples grown on the ground and in space under both the LD and the SD (Figure 4D). These results indicated that the number of genes differentially expressed in response to spaceflight was overall greater than the number of genes controlled by day length, suggesting a major impact of spaceflight on global transcription in the leaves of WT and FG plants under both LD and SD conditions.

**FIGURE 5:**
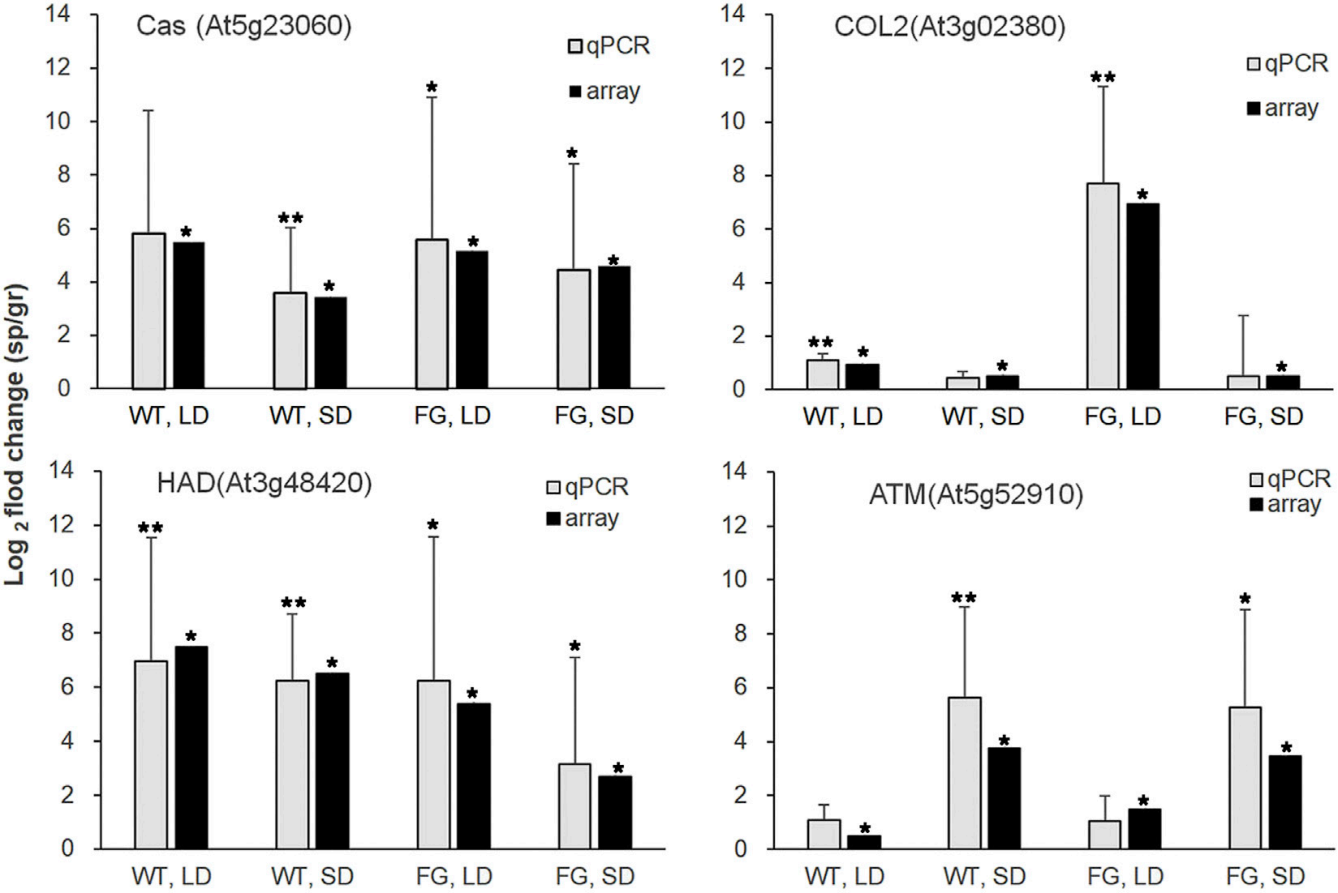
Relative transcript abundance changes in WT and FG under LD- and SD-conditions in space (sp) in comparison with the ground controls (gr), respectively, were analyzed by microarray (array) and real-time quantitative PCR (qPCR) for *Cas* (At5g23060), *HAD* (At3g48420), *COL2*(At3g02380) and *ATM*(At5g52910). Microarray data are shown as an average of two independent replicates, *p* values (*˂0.05) for microarray data (the significance of differences between sp and gr). qPCR with isoform-specific primers (Supplementary Table S1) for selected genes was performed on a third independent replicates. At3g18780 (*ACTIN*) was used as a quantitative control. Data are mean ± standard deviation, *p* values (*˂0.05, ** ˂0.01, student’s t-test) in qPCR.

To validate the microarray data, we generated sequence-specific primers and performed real-time quantitative RT-PCR on a third independent replicate. Real-time PCR with isoform specific primers for calcium sensing receptor (*Cas*, At5g23060), haloacid dehalogenase-like hydrolase superfamily protein (*HAD*, At3g48420), constans-like 2(*COL2*, At3g02380) and TIMELESS (*ATM*, At5g52910) confirmed the relative abundance changes for the transcript levels of these genes of WT and FG under LD and SD conditions in response to spaceflight (Figure 5).

### Daylength Related Spaceflight Responsive Genes

**FIGURE 6:**
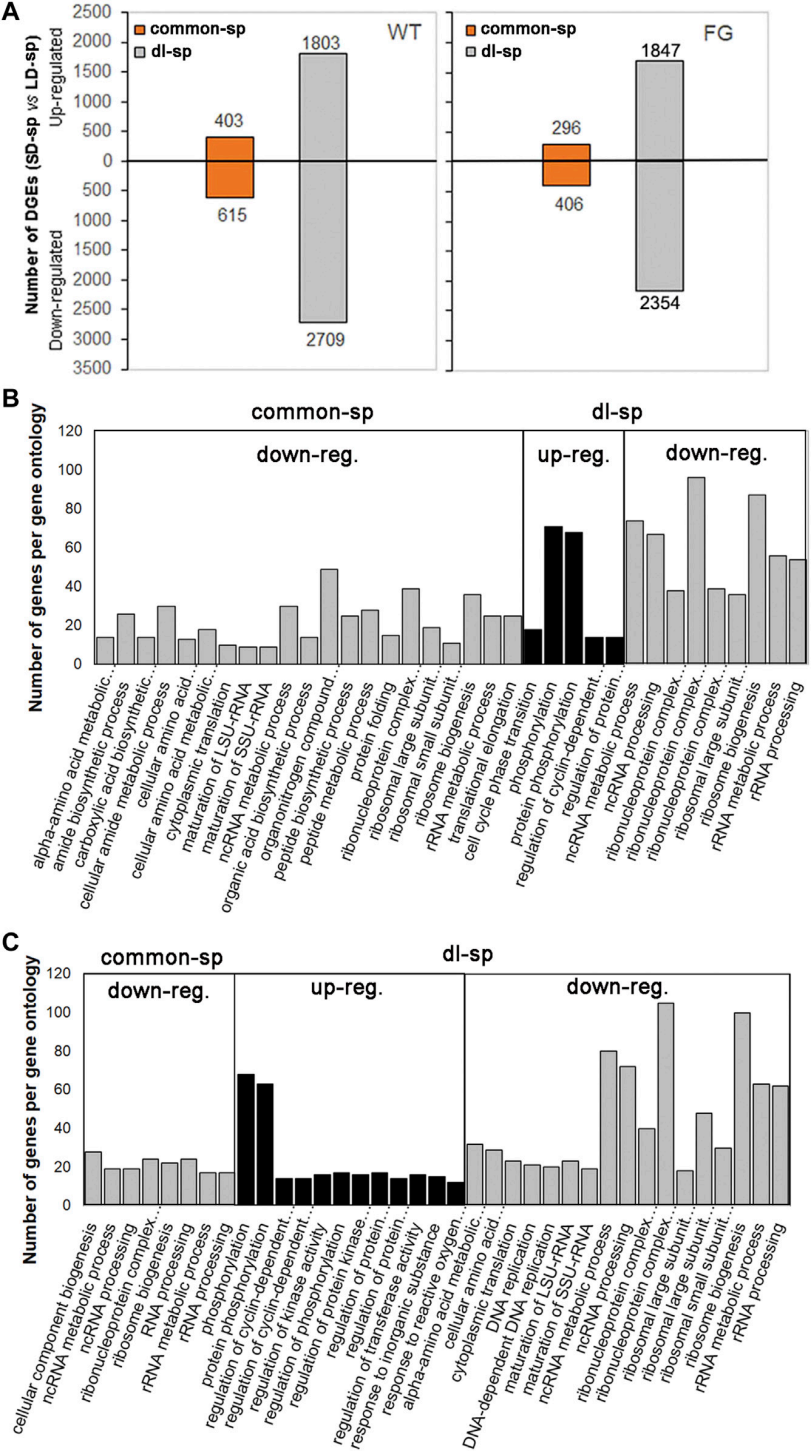
Pair-wise comparison and GO terms analysis of changes in spaceflight-affected gene expression. **(A)** Differential expression genes (DEGs) under the LD and the SD in space (sp) with similar behaviors as their controls on the ground (gr) under the same conditions (-1<log_2_FC (SD-sp/gr vs LD-sp/gr) < 1) are put the ‘common-sp’ group. DEGs under the SD in sp in comparison with their gr controls exhibited different levels from those under the LD in sp in comparison with their gr controls under the same condition (log_2_ FC (SD-sp/gr vs LD-sp/gr) > 1 or < -1) are put the ‘daylength-space related’ (dl-sp) group. FC, fold change. **(B,C)** GO terms overrepresented DEGs in the ‘common-sp’ and the ‘dl-sp’ groups of WT (B) and FG **(C)**. The significant gene ontology (PANTHER statitical overrepresentation test, GO-Slim biological precess, FDR *p*-value<0.01) categories from up-regulated (up-reg.) DEGs or down-regulated (down-reg.) DEGs in WT and FG under the LD and the SD are depicted. WT, wild-type; FG, *pHSP*:*FT*; *pHSP*:*GFP* trangenic plants.

To characterize the categories of gene expression affected by spaceflight under different photoperiods, two groups of genes were divided based on their transcriptional behaviors (Table 1). The genes responding to spaceflight under the LD and the SD conditions with similar behaviors were named ‘common-sp’ genes, whereas those in response to spaceflight specific to the LD or the SD were named ‘daylength-related-sp (dl-sp)’ genes. Among ‘common-sp’ genes, transcript levels of 1,018 genes in WT and 720 genes in FG showed similar changes when the plants were exposed to spaceflight under the LD and the SD (Figure 6A; Table 1; Supplementary Table S8 and S9). In contrast, a relatively large number of ‘dl-sp’ genes were found with total 4,512 genes in WT and 4,201 genes in FG (Table 1; Figure 6A; Supplementary Tables S8,9). Overall changes in gene expression pattern in WT and FG for ‘dl-sp’ genes were apparently more than those of ‘common-sp’, indicating that daylength is an important factor to regulate the response of plants to spaceflight. Gene Ontology (GO) categories representing ‘common-sp’ and ‘dl-sp’ genes in both WT and FG exhibited similar behaviors in the down-regulation of ribosome biogenesis and RNA processing (i.e. ncRNA, rRNA metabolism, and processing), amino acid metabolic process (Figures 6B,C). However, compared with ‘common-sp’ genes, significantly enriched GO terms were identified for ‘dl-sp’ genes, among which the most overrepresentation was protein phosphorylation GO category in both WT and FG (Figures 6B,C). The function of proteins encoded by these overrepresented ‘dl-sp’ protein phosphorylation genes are involved in light and ethylene signaling, calcium signaling, cell wall-associated receptor kinase-like proteins, phosphatase and protein kinase, and others (Supplementary Table S10).

**TABLE 1:** List of categories of differential expression genes affected by spaceflight under different photoperiods.

Group	Transcriptional behavior		Number of genes		Description
	LD-*sp*	SD-*sp*	WT	FG	
common-sp	YES	YES	1,018	702	Genes differentially expressed (DE) and their expression levels under SD-sp are similar to those under LD-sp
dl-sp	YES	YES	455	348	Genes DE under LD-sp and SD-sp, but with different behavior
	YES	NO	2,959	2,872	Genes DE only when sp is applied under LD
	NO	YES	1,098	981	Genes DE only when sp is applied under SD

**FIGURE 7:**
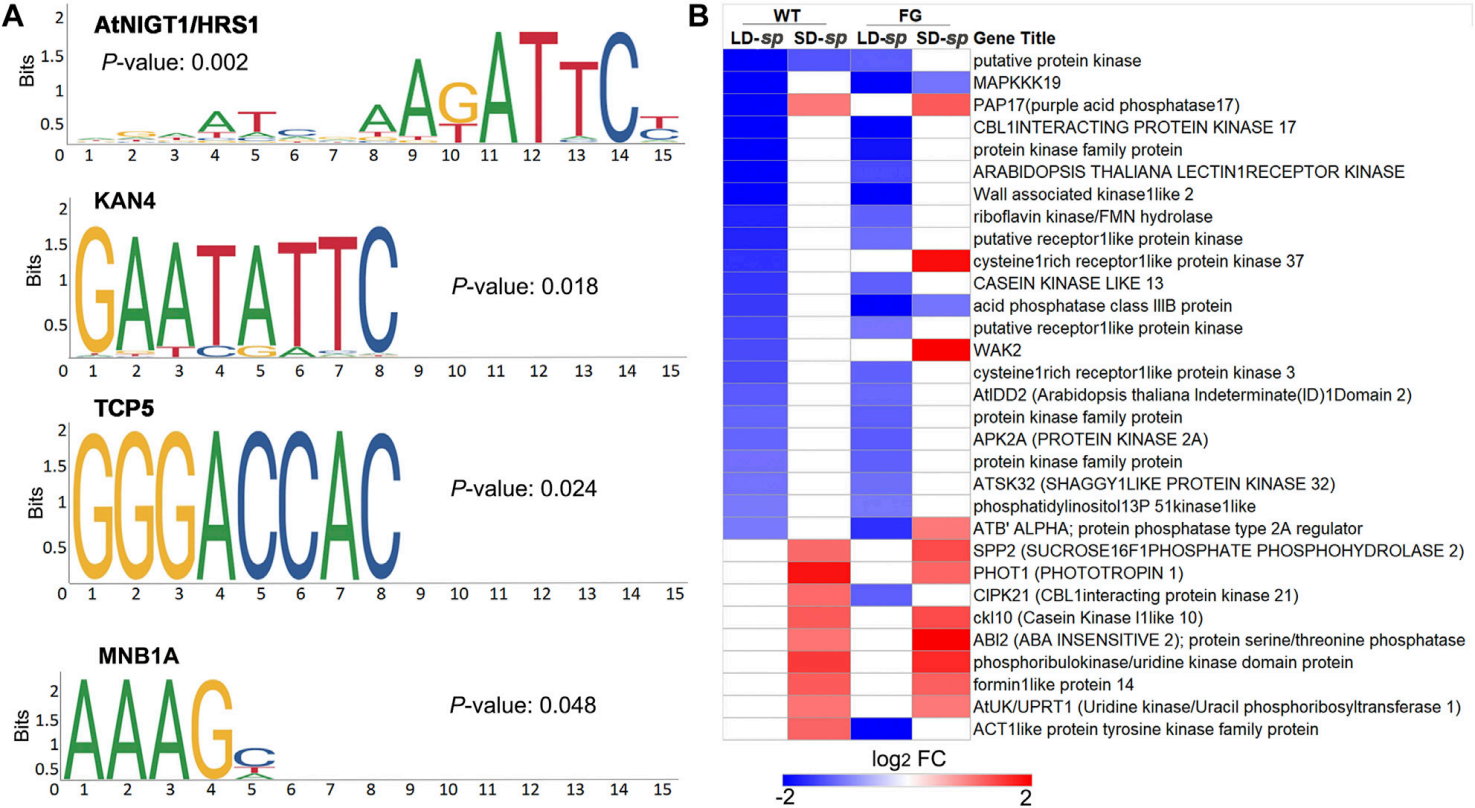
Potential regulator of co-regulated genes in protein phosphorylation functional cluster of ‘daylength-space related’ genes of WT and FG. **(A)** Four overreprented motifs enriched in upstream promoter sequences of genes in protein phosphorylation GO category in Figure 6B,C, as detected by the plant regulomics (bioinfo.sibs.ac.cn/plant-regulomics). Indicated are the *p*-Value representing the statistical significance of the motif. **(B)** Clustering analysis of the selected coregulated genes in protein phosphorylation GO category in Figures 6B,C, which changed transcript abundance in response to spaceflight in WT and FG plants under long-day (LD) and short-day (SD) conditions, respectively and have AtNIGT1/HRS1, KAN4, TCP five and MNB1A binding sites in upstream promoter regions. LD-*sp*, Genes of plants under the LD differentially expressed in space in comparison with their controls on ground. SD-*sp* **,** Genes of plants under the SD differentially expressed in space in comparison with their controls on ground.

Protein phosphorylation is one of the processes involved in regulating plant stress responses (Barjaktarović et al., 2009; Yin et al., 2018). To test whether there are potential common *cis*-acting elements among these ‘dl-sp’ protein phosphorylation genes, we performed analyses using Plant Regulomics (bioinfo.sibs.ac.cn/plant-regulomics) to find overrepresented motifs in the 1-kb upstream sequence of the overrepresented genes. Thirty-one coregulated genes were identified to share four common motifs, of which occurrences were significantly high as compared to that in random genomic regions (Figures 7A,B). These include A (G/T)ATTC, which is identical to the AtNIGT1/HRS1 (AT1G13300) motif that is present in the promoters of nitrate and phosphate signaling genes (Medici et al., 2015), GAATATTC, which represents the KAN4 (AT5G42630) motif that provides boundary maintenance and promotes the laminar growth of the inner ovule integument (Gomez et al., 2016), and GGGACCAC, which is identical to the transcription factor TCP5 (AT5G60970) that controls plant thermo-morphogenesis by positively regulating PHYTOCHROME INTERACTING FACTOR 4(PIF4) activity (Han et al., 2019). Furthermore, the AAAG, which is similar to the dof zinc finger protein MNB1A element that has been suggested to regulate photosynthetic gene expression in *Zea may* (Cavalar et al., 2006). This result indicated that common regulators were involved in adaptation of plants to daylength-related spaceflight response.

### Expression of *FT* Affect Daylength-Related Spaceflight Response

**FIGURE 8:**
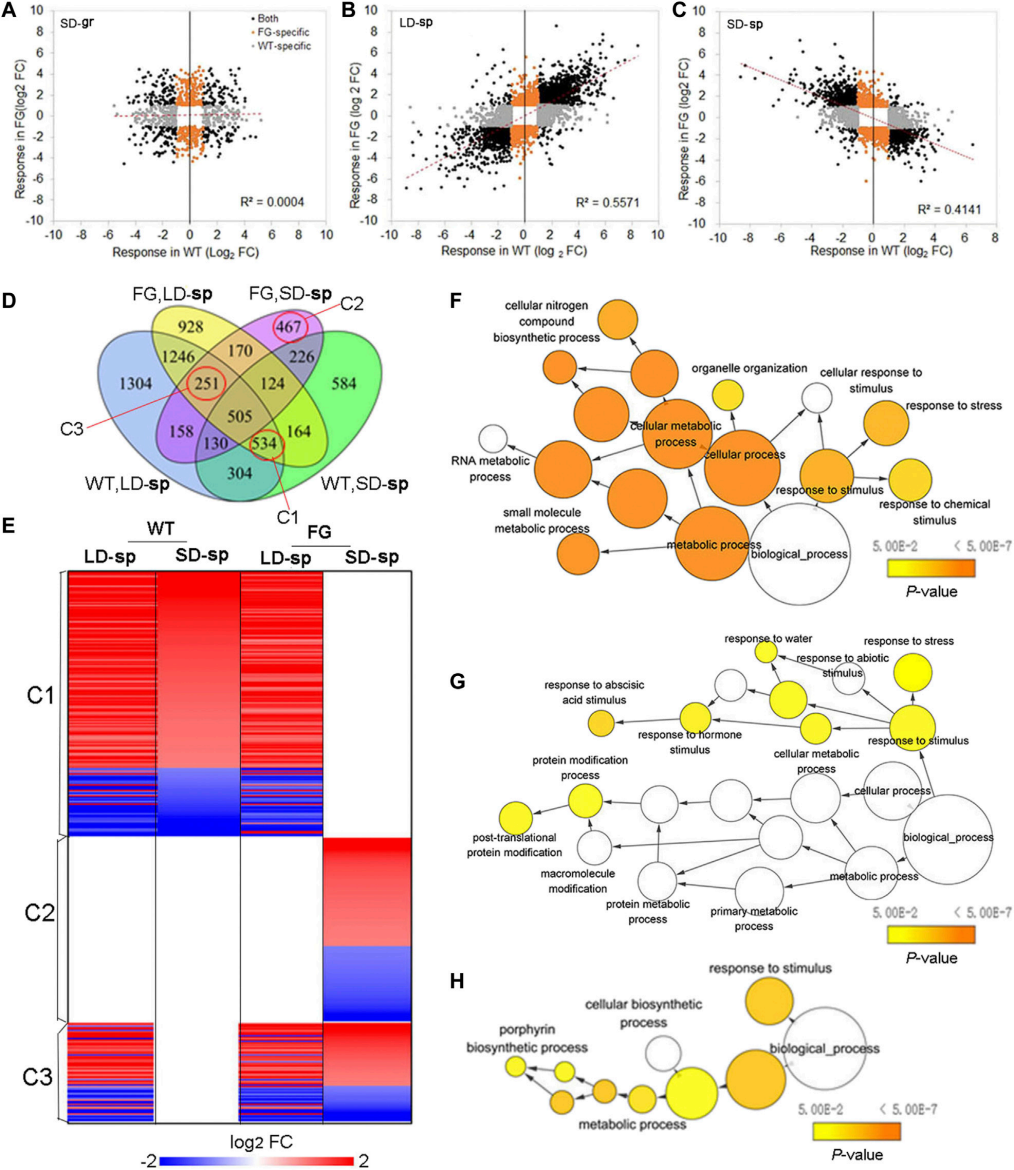
Expression of *FT* affected photoperiod-spaceflight response transcriptome. **(A–C)** Scatterplot showing the spaceflight response of differential expression genes (DEGs) between FG and WT under the LD and the SD, respectively. **(D)** Venn diagrams summarizing the number of DEGs in response to spaceflight under the LD and the SD, respectively, among WT and FG samples (FC > 2 and *p* < 0.05). DEGs in cluster 1(C1), C2 and C3 marked with red circles were selected to further analyze. FC, fold change. **(E)** Selected DEGs in FG plants in response to spaceflight under the LD (LD-sp) and the SD (SD-sp) are compared with those in WT plants in space. C1, C2 and C3 are indicated in D. **(F–H)** Enriched GO Terms in DEGs of selected clusters in D. The networks graphs show BiNGO visualization of the overrepresented GO terms for the selected clusters corresponding to cluster C1 to C3 indicating in D and E. Categories in GoslimPlants were used to simplify this analysis. Uncolored nodes are not overrepresented, but they may be the parents of overrepresented terms. Colored nodes represent GO terms that are significantly overrepresented (Benjamini and Hochberg corrected *p* value < 0.05), with the shade indicating significance as shown in the color bars.

Transcriptional control of *FT* expression is an important strategy for plants to cope with environmental stresses (Riboni et al., 2013; Kazan and Lyons, 2016). To know whether altered expression of *FT* could also affect the response of plants to the spaceflight stress, we compared expression of ‘dl-sp’ genes in FG with those in WT. The directional comparison of DEGs in FG with those in WT under the SD on the ground showed no significant correlation (Figure 8A). In contrast, a comparison of DEGs in FG with those in WT under the LD in space showed a strong positive correlation (Figure 8B), while DEGs in FG under the SD in space showed a strong negative correlation with those in WT under the same condition (Figure 8C), suggesting that the response of FG to spaceflight was similar to those in WT under the LD, but different under the SD, when *FT* had expressed in FG plants by heating induction.

To determine whether any GO was significantly enriched in FG after the induction of *FT* expression under the SD in spaceflight in comparison with WT under the same condition, GO classifications of DEGs (Figure 8C) were performed using PANTHE (http://www.pantherdb.org). The statistical overrepresentation test revealed 58 biological processes, eight protein class, 18 cellular components, and 24 molecular functions showing statistical overrepresentation (Supplementary Table S11). Regarding biological processes, cellular process and metabolic process each accounted for 37 and 31% of gene classifications, accounting for the majority of DEGs (Supplementary Figure S3A), followed by biological regulation (11.6%), response to stimulus (8.7%), localization (4.8), and signaling (2.7%). The gene classification of molecular function in FG under SD affected by spaceflight after induction of FT expression was also conducted. Catalytic activity and binding accounted for 49% and 30% of DEGs, respectively, while molecular function regulator and transporter activity accounted for 7.6% and 7.4%, respectively (Suppl. Fig. 3SB). Smaller contributing ontologies included structural molecule activity (4.0%) and molecular transducer activity (1.0%). Further division into categories of these ontologies is provided in Supplementary Figure S3 and Supplementary Table S11.

To further study response of plants to spaceflight after *FT* expression in FG under the SD in comparison with WT, we focused on DEGs in three clusters (Figure 8D, Supplementary Table S12). Cluster 1(C1) comprises 534 DEGs, of which expression in WT under both the LD and the SD in space as well as in FG under the LD in space were modified, but unchanged in FG under the SD in space in comparison with their controls on the ground (Figures 8D,E). Thus, DEGs of C1 represent the group of genes in FG under the SD condition insensitive to spaceflight stress in comparison with those in WT under the same condition. Analysis of this cluster to assess overrepresented GO terms with the Biological Networks Gene Ontology tool (BINGO) indicated a link with cellular metabolic processes, notably amino acid, amine, and oxoacid metabolic process (Figure 8F). Cluster 2(C2) is represented by 467 DEGs. Their expression did not change in WT under the LD and the SD as well in FG under the LD in space but was significantly up-or down-regulated in FG under the SD in space in comparison with their controls on the ground. In contrast to C1, C2 represents those more sensitive to spaceflight in FG under the SD condition. BINGO analysis of the DEGs revealed that this cluster included many of the abiotic stimulus response-associated genes noted in Figure 8G, as well as a number of genes involved in post-translational protein modification. Cluster 3 (C3) include 251 DEGs, which altered expression in response to spaceflight in both WT and FG under the LD as well as in FG under the SD, but which did not change expression level in WT under the SD. The DEGs in this cluster represent the LD-sp response which was activated in FG under the SD condition. BINGO analysis of this cluster of DEGs suggested that the activated processes include metabolic processes, notably a porphyrin biosynthetic process in the chlorophyll biosynthetic pathway, and response to stimulus (Figure 8H; Supplementary Table S12). These results indicated that expression of *FT* in FG plants by HS treatment could alter plant response to spaceflight, possibly through change sensitivity to the abiotic stimulus and/or cellular metabolic process.

### Impact of Spaceflight on Daylength Flowering Pathways

**FIGURE 9:**
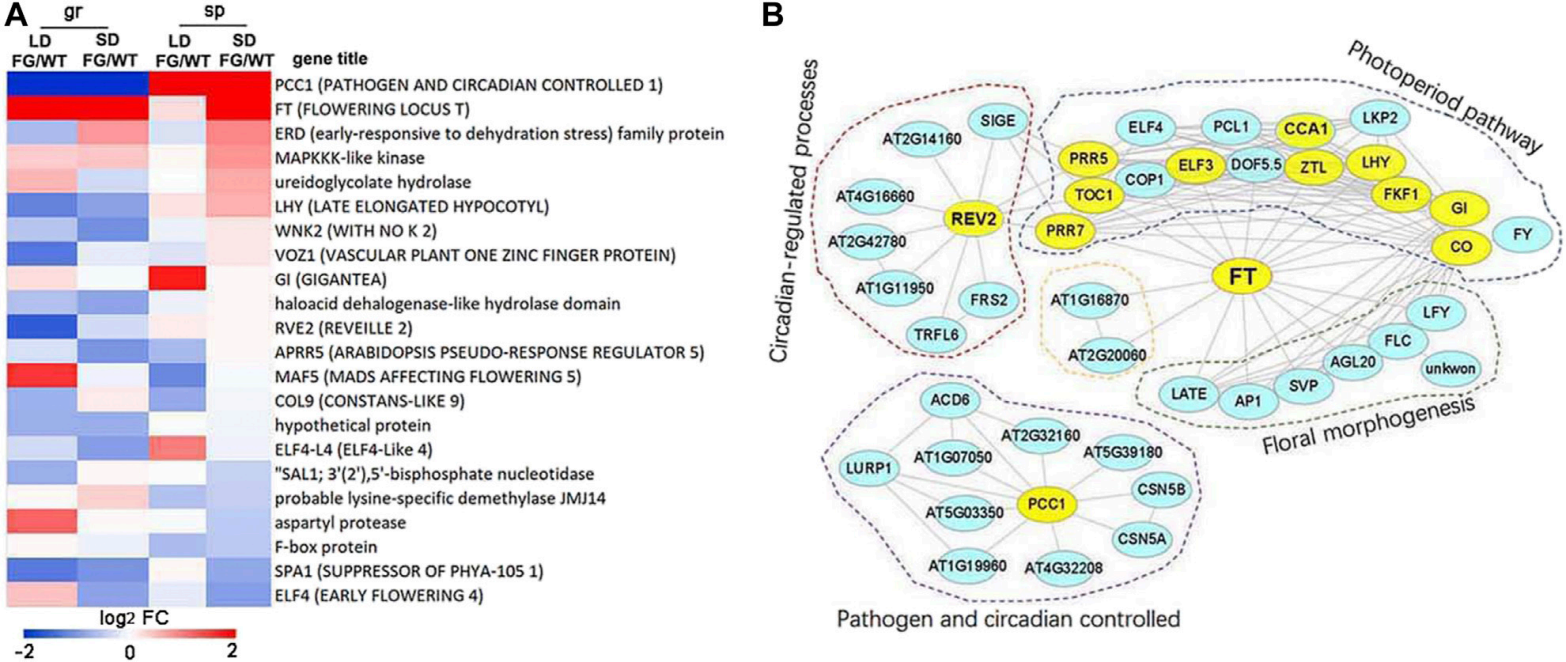
The core photoperiod responsive genes altered expression levels by exposuree to spaceflight. **(A)** Log_2_ flod change of the 20 core photoperiod genes in the FG under LD or SD on ground (gr) and under spaceflight (sp), respectively, in comparison with these genes in wild-type (WT) under the same condition (FG/WT). **(B)** Diagram of the FT protein interaction networks of the photoperiod responsive genes. The genes, which changed expression levels in response to spaceflight in comparison with their controls on ground, were labelled by color in yellow.

To further explore the impact of spaceflight on daylength flowering pathways, we investigated the expression pattern of the 49 core flowering control genes (http://wikipathways.org) in response to spaceflight in WT and FG under the LD and the SD, respectively. Thirty-seven of them showed altered expression levels in response to spaceflight in WT and/or FG under at least one of the daylength conditions (Supplementary Table S13). More than one-third of these core flowering control genes are circadian clock genes, including, *LHY* (AT1G01060), *RVE1* (AT5G17300), *RVE2* (AT5G37260), *CCA1* (AT2G46830), *PCC1* (AT3G22231), *ELF4* (AT2G40080), *ELF4-L4* (AT1G17455), *GI* (AT1G22770), *COP1* (AT2G32950), *CO* (AT5G15840), *COL9* (AT3G07650), and *APRR5* (AT5G24470). The differential expressions of these core flowering control genes between WT and FG in response to spaceflight was observed (Figure 9A). For example, expression of *PCC1* was down-regulated in FG on the ground, while up-regulated in space in comparison with that in WT. Expression of *FT* in FG was apparently higher under both LD and SD conditions on the ground and in space than that in WT (Figure 9A), consistent with GFP signal in FG observed by inflight images (Figure 3P). In addition, *ERD*, MAPKKK-like kinase, ureidoglyconlate hydrolase, and *LHY* were up-regulated, while *ELF4*, *SPA1,* and F-box protein were down-regulated in FG under the SD condition in space in comparison to those in WT under the same conditions (Figure 9A).

Furthermore, several circadian clock genes were observed among FT interactome (Figure 9B). *REV2*, which is involved in regulating both photoperiod pathways and circadian processes, was up-regulated about 115-fold in FG and about 35-fold in WT under the LD in space in comparison with their control on the ground, but less increased under the SD (Supplementary Table S13). Expression level of *LHY* exhibited up-regulation under LD in WT and FG in space (5.82- and 15.56-fold, respectively), but more significantly increased under SD in space (9.05- and 26-fold, respectively). In contrast, expression level of *GI* was down-regulated under both LD and SD in WT and FG during spaceflight (Supplementary Table S13). These results indicated that circadian clock genes could play an important role in FT integrated daylength pathways for plant adaptation to spaceflight at the reproductive developmental stage.

## Data Availability

The data presented in the study are deposited in the (https://www.ncbi.nlm.nih.gov/geo) repository, accession number (GSE190748).

## Discussion

Day length and the spaceflight condition both impact the growth and development of plants, but interaction between them remains unclear. In this study, we constructed a transgenic plant (*pHSP:FT*; *pHSP:GFP*) and designed a protocol for the induction of FT expression on orbital condition using HS treatment by remote control. Using this experimental system, we revealed: 1) plant response to spaceflight at transcriptional levels is associated with the daylength conditions. 2) daylength-related spaceflight response could be involved in the alteration of transcriptional activity in protein synthesis and post-translation protein modulation, notably protein phosphorylation. 3) Expression of *FT* in Arabidopsis leaves by HS induction under the SD condition in space can alter the expression of gene function in the cellular metabolic process and/or the abiotic stimulus.

Genetic manipulation of gene expression at key developmental stage using a gene switch, which can mediate an “on/off” situation of gene activity, will be very important to design efficient production and resilient crop cultivation in a bioregenerative life-support system (BLSS) for a long-term mission such as on an earth-orbit space station, the Moon, and Mars in the future. A previous study using transgenic Arabidopsis containing the alcohol dehydrogenase (Adh) gene promoter linked to the *β*-glucuronidase (GUS) reporter gene has been used to evaluate the stress signal perception and transduction in Arabidopsis during spaceflight (Paul et al., 2001). In this study, we demonstrated that a *HSP17.4* promoter combined with *FT* and *GFP* could be used in the flowering induction of transgenic Arabidopsis by simply heating the culture chamber (37°C, 1 h), and expression of *GFP* gene in leaves, which could be directly monitored by a remote camera. However, HSP17.4, which belongs to a small heat shock protein family, is synthesized in response to high temperatures and other stresses in the vegetative tissues of plants (Ré et al., 2017). In our study, FG plants showed slight earlier bolting under LD (without HS) in both ground and space conditions indicating activation of *HSP17.4* promoter in plants induced in advance possibly by a stress caused by closed culture condition of PGU before the HS was carried out. However, it is no problem for the goal of this study, which was not only to study the effect of FT on flowering induction, but also the effects of FT expression on the reproductive developmental stage after flowering. In addition, the GFP signals of FG under the SD displayed wave-shape (Figure 3P) changed could be due to the temperature of plant leaves in light (about 27°C) and in dark (about 20°C) in the culture chambers and *HSP17.4* could be very sensitive to the change of temperature. Nonetheless, this system provided a possible approach to manipulating crops for high production in space through controlling plant reproductive development.

Flowering is a prerequisite for crop production whenever seeds or fruits are harvested (Blümel et al., 2015; Shim et al., 2017). Various environmental stresses, such as drought, salt and temperature, were reported to have interfered with flowering (Galbiati et al., 2016; Fernández et al., 2016). Spaceflight is a novel stress to plants, which evolved on Earth (Hampp et al., 1997; Zheng et al., 2008; Zhang and Zheng, 2015; Paul et al., 2017; Wu et al., 2020). Previous studies indicated that spaceflight is a compound stress, imposing multiple constraints on plants by integration of spaceflight conditions. For example, roots of plants in space appeared to become hydrotropically more sensitive to moisture gradients (Morohashi et al., 2017) and develop an altered response to red- and blue-light phototropism (Valbuena et al., 2018; Herranz et al., 2019). In this study, we found that Arabidopsis plants grown under the LD were more sensitive to spaceflight in comparison those under the SD condition at a transcriptional level. Down-regulation of expression in ribosome biogenesis and RNA processing (i.e. ncRNA, rRNA metabolism, and processing) and amino acid metabolic process was observed among ‘common-sp’ genes as well as ‘dl-sp’ genes. This result is consistent with the fact that regulation of ribosome biogenesis, which is linked to factors controlling cell growth and proliferation, was decreased in Arabidopsis cell cultures and seedlings in real microgravity in space or simulated microgravity on the ground (Matía et al., 2010; Manzano et al., 2012; Kamal et al., 2018). The reason for down-regulation of ribosome biogenesis under spaceflight is unknown. A previous study estimated that cells dedicate ∼80% of total transcriptional activity to the synthesis of rRNAs and proteins for ribosome biogenesis (Warner, 1999), making ribosome biogenesis a major nutrient and energy-consuming process in growing cells (Lempiäinen and Shore, 2009). Down-regulation of ribosome biogenesis found in ‘common-sp’ genes suggest a limitation in nutrient and energy supply to plants during the spaceflight response under both the LD and the SD condition. Another interesting finding is our observation that spaceflight trigger networks related to daylength signals altered expression of genes involved in the GO category ‘protein phosphorylation’ in both the WT and FG. These ‘dl-sp’ genes, including protein kinase, receptor-like protein kinase, phosphatase, and signaling in light, ethylene, and calcium, were down-regulated expression in the LD-sp datasets and/or up-regulated expression in the SD-sp datasets. Among ‘protein phosphorylation’ GO category genes, we found phytochrome interacting factor 4(*PIF4*) and phototropic-responsive *NPH3* family protein, and a blue light receptor phototropin 1(*PHOT1*), which showed that expression profiles in response to spaceflight were modulated differently by day length. Similarly, expression of genes involved in sensing the extracellular environment and triggering intracellular signals, such as several wall-associated kinases (WAK), ethylene response sensors, and calcium-dependent protein kinases, were mostly downregulated in response to spaceflight under the LD only, but did not change expression level under the SD.

Plant photoperiodic regulation can be divided into three parts: light input, circadian clock, and output. A previous study pointed out that light-associated pathways in Arabidopsis showed significant down-regulation during spaceflight (Valbuena et al., 2018; Vandenbrink et al., 2019). However, those experiments were all performed under the LD condition, but not under the SD. Our study found that light-associated genes, such as *PHOT1,* were down-regulated only under the LD, while expression of *PIF4* and *NPH3* did not change under the LD, but up-regulated specifically under the SD under spaceflight, suggesting that photoperiod apparently affected the plant’s response to spaceflight during light input. In addition, light information is integrated into innate photoperiodic timing mechanisms governed by the circadian clock to induce *FT* expression that triggers flowering (Shim et al., 2017). The plant circadian clock consists of multiple transcription-translation feedback loops that are influenced by environmental signals, linking the clock with plant stress adaptation. The expression of circadian clock components, such as *CCA1*, *ELF3*, *GI*, *GRP7*, *PRR9*, TOC1, and ZTL, were affected by stress environment, including water use efficiency of Arabidopsis (Simon et al., 2020), high light (Yakir et al., 2007) as well as the 3-D clinostat rotational simulated microgravity on the ground (Xie and Zheng, 2020). In this study, our data indicate that the circadian oscillator is important for regulating the spaceflight response of Arabidopsis. Up-regulation of morning components (*CCA1*, *LHY*, *REV1,* and *REV2*) and down-regulation of late day (*GI*) and evening (*ELF4-L4*) components of the circadian oscillator in space under the LD and/or the SD in both WT and FG. Additionally, *COL9* and *COP1* enhanced expression levels in response to spaceflight specifically under the LD condition, while *APRR5* increased expression levels in response to spaceflight specifically under the SD condition. Together, these results suggest that the circadian oscillators can be altered by expression levels in response to spaceflight dependent on daylength condition, which could interfere with flowering in space. Changing the circadian function by influencing plant flowering and fitness in spaceflight offers the hypothesis that optimizing circadian function will enhance crop productivity in space.

In addition, the plants for transcription analysis in this study were sampled about 2 h post-landing, the spaceflight responsive DEGs we identified above only represent those spaceflight adaptation genes, the expression behaviors of which were not recovered by the short-term altered gravity during landing. Many other spaceflight responsive genes remain to be identified. These include those short-term altered gravity responsive genes, such as those which function by dramatically increasing production of ROS and other radicals in plants during short-term gravity stimulus (Babbick et al., 2007). In the future, more refinement of our understanding on the response of plants to spaceflight conditions, including microgravity, is necessary to inform manipulation towards the goal of enhancing crop productivity in BLSS (Czarnecka et al., 1990; Wehmeyer et al., 1996).

## References

[B1] Achard P. Cheng H. De Grauwe L. Decat J. Schoutteten H. Moritz T. (2006). Integration of Plant Responses to Environmentally Activated Phytohormonal Signals. Science 311, 91–94. 10.1126/science.1118642 16400150

[B2] Babbick M. Dijkstra C. Larkin O. J. Anthony P. Davey M. R. Power J. B. (2007). Expression of Transcription Factors after Short-Term Exposure of *Arabidopsis thaliana* Cell Cultures to Hypergravity and Simulated Microgravity (2-D/3-D Clinorotation, Magnetic Levitation). Adv. Space Res. 39, 1182–1189. 10.1016/j.asr.2007.01.001

[B3] Barjaktarovic Z. Schütz W. Madlung J. Fladerer C. Nordheim A. Hampp R. (2009). Changes in the Effective Gravitational Field Strength Affect the State of Phosphorylation of Stress-Related Proteins in Callus Cultures of *Arabidopsis thaliana* . J. Exp. Bot. 60, 779–789. 10.1093/jxb/ern324 19129159 PMC2652066

[B4] Becker B. Holtgrefe S. Jung S. Wunrau C. Kandlbinder A. Baier M. (2005). Influence of the Photoperiod on Redox Regulation and Stress Responses in *Arabidopsis thaliana* L. (Heynh.) Plants under Long- and Short-Day Conditions. Planta 224, 380–393. 10.1007/s00425-006-0222-3 16435132

[B5] Blümel M. Dally N. Jung C. (2015). Flowering Time Regulation in Crops - what Did We Learn from Arabidopsis. Curr. Opin. Biotechnol. 32, 121–129. 10.1016/j.copbio.2014.11.023 25553537

[B6] Boyes D. C. Zayed A. M. Ascenzi R. McCaskill A. J. Hoffman N. E. Davis K. R. (2001). Growth Stage-Based Phenotypic Analysis of Arabidopsis. Plant Cell 13, 1499–1510. 10.1105/tpc.010011 11449047 PMC139543

[B7] Campbell W. F. Salisbury F. B. Bugbee B. Klassen S. Naegle E. Strickland D. T. (2001). Comparative floral Development of Mir-Grown and Ethylene-Treated, Earth-Grown Super Dwarf Wheat. J. Plant Physiol. 158, 1051–1060. 10.1078/s0176-1617(04)70129-7 12033229

[B8] Cavalar M. Phlippen Y. Kreuzaler F. Peterhänsel C. (2006). A Drastic Reduction in DOF1 Transcript Levels Does Not Affect C4-specific Gene Expression in maize. J. Plant Physiol. 164, 1665–1674. 10.1016/j.jplph.2006.09.008 17178169

[B9] Czarnecka E. Fox P. C. Gurley W. B. (1990). *In Vitro* interaction of Nuclear Proteins with the Promoter of Soybean Heat Shock Gene Gmhsp17.5E. Plant Physiol. 94, 935–943. 10.1104/pp.94.3.935 16667874 PMC1077325

[B10] De Micco V. De Pascale S. Paradiso R. Aronne G. (2014). Microgravity Effects on Different Stages of Higher Plant Life Cycle and Completion of Theseed-To-Seedcycle. Plant Biol. J. 16, 31–38. 10.1111/plb.12098 24015754

[B11] Fernández V. Takahashi Y. Le Gourrierec J. Coupland G. (2016). Photoperiodic and Thermosensory Pathways Interact through CONSTANS to Promote Flowering at High Temperature under Short Days. Plant J. 86, 426–440. 10.1111/tpj.13183 27117775

[B12] Galbiati F. Chiozzotto R. Locatelli F. Spada A. Genga A. Fornara F. (2016). Hd3a,RFT1andEhd1integrate Photoperiodic and Drought Stress Signals to Delay the floral Transition in rice. Plant Cel Environ. 39, 1982–1993. 10.1111/pce.12760 27111837

[B13] Gomez M. D. Ventimilla D. Sacristan R. Perez-Amador M. A. (2016). Gibberellins Regulate Ovule Integument Development by Interfering with the Transcription Factor ATS. Plant Physiol. 172, 2403–2415. 10.1104/pp.16.01231 27794102 PMC5129715

[B14] Hampp R. Hoffmann E. Schönherr K. Johann P. Filippis L. D. (1997). Fusion and Metabolism of Plant Cells as Affected by Microgravity. Planta 203, S42–S53. 10.1007/pl00008114 9299795

[B15] Han X. Yu H. Yuan R. Yang Y. An F. Qin G. (2019). Arabidopsis Transcription Factor TCP5 Controls Plant Thermomorphogenesis by Positively Regulating PIF4 Activity. iScience 15, 611–622. 10.1016/j.isci.2019.04.005 31078552 PMC6548983

[B16] Herranz R. Vandenbrink J. P. Villacampa A. Manzano A. Poehlman W. L. Feltus F. A. (2019). RNAseq Analysis of the Response of *Arabidopsis thaliana* to Fractional Gravity under Blue-Light Stimulation during Spaceflight. Front. Plant Sci. 10, 1529. 10.3389/fpls.2019.01529 31850027 PMC6889863

[B17] Hoson T. Soga K. Wakabayashi K. Hashimoto T. Karahara I. Yano S. (2014). Growth Stimulation in Inflorescences of anArabidopsistubulin Mutant under Microgravity Conditions in Space. Plant Biol. J. 16, 91–96. 10.1111/plb.12099 24148142

[B18] Kamal K. Y. Herranz R. van Loon J. J. W. A. Medina F. J. (2018). Simulated Microgravity, Mars Gravity, and 2g Hypergravity Affect Cell Cycle Regulation, Ribosome Biogenesis, and Epigenetics in Arabidopsis Cell Cultures. Sci. Rep. 8, 6424. 10.1038/s41598-018-24942-7 29686401 PMC5913308

[B19] Karahara I. Suto T. Yamaguchi T. Yashiro U. Tamaoki D. Okamoto E. (2020). Vegetative and Reproductive Growth of Arabidopsis under Microgravity Conditions in Space. J. Plant Res. 133, 571–585. 10.1007/s10265-020-01200-4 32424466

[B20] Kazan K. Lyons R. (2016). The Link between Flowering Time and Stress Tolerance. J. Exp. Bot. 67, 47–60. 10.1093/jxb/erv441 26428061

[B21] Kiss J. Z. Brinckmann E. Brillouet C. (2000). Development and Growth of Several Strains of Arabidopsis Seedlings in Microgravity. Int. J. Plant Sci. 161, 55–62. 10.1086/314223 10648194

[B22] Krikorian A. D. O'Connor S. A. (1984). Karyological Observations. Ann. Bot. 54, 49–63. 10.1093/oxfordjournals.aob.a086866 11538823

[B23] Kuang A. Musgrave M. E. Matthews S. W. (1996). Modification of Reproductive Development in *Arabidopsis thaliana* under Spaceflight Conditions. Planta 198, 588–594. 10.1007/bf00262646 11539321

[B24] Kwon T. Sparks J. A. Nakashima J. Allen S. N. Tang Y. Blancaflor E. B. (2015). Transcriptional Response of Arabidopsis Seedlings during Spaceflight Reveals Peroxidase and Cell wall Remodeling Genes Associated with Root Hair Development. Am. J. Bot. 102, 21–35. 10.3732/ajb.1400458 25587145

[B25] Lempiäinen H. Shore D. (2009). Growth Control and Ribosome Biogenesis. Curr. Opin. Cel Biol. 21, 855–863. 10.1016/j.ceb.2009.09.002 19796927

[B26] Levinskikh M. A. Sychev V. N. Derendyaeva T. A. Signalova O. B. Salisbury F. B. Campbell W. F. (2000). Analysis of the Spaceflight Effects on Growth and Development of Super dwarf Wheat Grown on the Space Station Mir. J. Plant Physiol. 156, 522–529. 10.1016/s0176-1617(00)80168-6 11543345

[B27] Link B. M. Busse J. S. Stankovic B. (2014). Seed-to-seed-to-seed Growth and Development of Arabidopsis in Microgravity. Astrobiology 14, 866–875. 10.1089/ast.2014.1184 25317938 PMC4201294

[B28] Link B. M. Durst S. J. Zhou W. Stankovic B. (2003). Seed-to-seed Growth of *Arabidopsis thaliana* on the International Space Station. Adv. Space Res. 31, 2237–2243. 10.1016/s0273-1177(03)00250-3 14686438

[B29] Ma Y. Shabala S. Li C. Liu C. Zhang W. Zhou M. (2015). Quantitative Trait Loci for Salinity Tolerance Identified under Drained and Waterlogged Conditions and Their Association with Flowering Time in Barley (*Hordeum Vulgare.* L). PLoS One 10, e0134822. 10.1371/journal.pone.0134822 26247774 PMC4527667

[B30] Manzano A. I. van Loon J. J. Christianen P. C. Gonzalez-Rubio J. M. Medina F. J. Herranz R. (2012). Gravitational and Magnetic Field Variations Synergize to Cause Subtle Variations in the Global Transcriptional State of Arabidopsis *In Vitro* Callus Cultures. BMC Genomics 13, 105. 10.1186/1471-2164-13-105 22435851 PMC3368779

[B31] Matía I. González-Camacho F. Herranz R. Kiss J. Z. Gasset G. van Loon J. J. W. A. (2010). Plant Cell Proliferation and Growth Are Altered by Microgravity Conditions in Spaceflight. J. Plant Physiol. 167, 184–193. 10.1016/j.jplph.2009.08.012 19864040

[B32] Medici A. Marshall-Colon A. Ronzier E. Szponarski W. Wang R. Gojon A. (2015). AtNIGT1/HRS1 Integrates Nitrate and Phosphate Signals at the Arabidopsis Root Tip. Nat. Commun. 6, 6274. 10.1038/ncomms7274 25723764 PMC4373655

[B33] Mi H. Muruganujan A. Huang X. Ebert D. Mills C. Guo X. (2019). Protocol Update for Large-Scale Genome and Gene Function Analysis with the PANTHER Classification System (v.14.0). Nat. Protoc. 14, 703–721. 10.1038/s41596-019-0128-8 30804569 PMC6519457

[B34] Morohashi K. Okamoto M. Yamazaki C. Fujii N. Miyazawa Y. Kamada M. (2017). Gravitropism Interferes with Hydrotropism via Counteracting Auxin Dynamics in Cucumber Roots: Clinorotation and Spaceflight Experiments. New Phytol. 215, 1476–1489. 10.1111/nph.14689 28722158

[B35] Murashige T. Skoog F. (1962). A Revised Medium for Rapid Growth and Bio Assays with Tobacco Tissue Cultures. Physiol. Plant 15, 473–497. 10.1111/j.1399-3054.1962.tb08052.x

[B36] Paul A.-L. Daugherty C. J. Bihn E. A. Chapman D. K. Norwood K. L. L. Ferl R. J. (2001). Transgene Expression Patterns Indicate that Spaceflight Affects Stress Signal Perception and Transduction in Arabidopsis. Plant Physiol. 126, 613–621. 10.1104/pp.126.2.613 11402191 PMC111153

[B37] Paul A.-L. Sng N. J. Zupanska A. K. Krishnamurthy A. Schultz E. R. Ferl R. J. (2017). Genetic Dissection of the Arabidopsis Spaceflight Transcriptome: Are Some Responses Dispensable for the Physiological Adaptation of Plants to Spaceflight. PLoS One 12, e0180186. 10.1371/journal.pone.0180186 28662188 PMC5491145

[B38] Paul A.-L. Zupanska A. K. Ostrow D. T. Zhang Y. Sun Y. Li J.-L. (2012). Spaceflight Transcriptomes: Unique Responses to a Novel Environment. Astrobiology 12, 40–56. 10.1089/ast.2011.0696 22221117 PMC3264962

[B39] Qi B. Zheng H. (2013). Modulation of Root-Skewing Responses byKNAT1inArabidopsis Thaliana. Plant J. 76, 380–392. 10.1111/tpj.12295 23889705

[B40] Ran X. Zhao F. Wang Y. Liu J. Zhuang Y. Ye L. (2019). Plant Regulomics: a Data-Driven Interface for Retrieving Upstream Regulators from Plant Multi-Omics Data. Plant J. 101, 237–248. 10.1111/tpj.14526 31494994

[B41] Rasmussen O. Baggerud C. A. Larssen H. C. Evjen K. Iversen T.-H. (1994). The Effect of 8 Days of Microgravity on Regeneration of Intact Plants from Protoplasts. Physiol. Plant 92, 404–411. 10.1034/j.1399-3054.1994.920306.x

[B42] Ré M. D. Gonzalez C. Escobar M. R. Sossi M. L. Valle E. M. Boggio S. B. (2017). Small Heat Shock Proteins and the Postharvest Chilling Tolerance of Tomato Fruit. Physiol. Plantarum 159, 148–160. 10.1111/ppl.12491 27545651

[B43] Riboni M. Galbiati M. Tonelli C. Conti L. (2013). GIGANTEA Enables Drought Escape Response via Abscisic Acid-dependent Activation of the Florigens and SUPPRESSOR of OVEREXPRESSION of CONSTANS1. Plant Physiol. 162, 1706–1719. 10.1104/pp.113.217729 23719890 PMC3707542

[B44] Sherrard M. E. Maherali H. (2006). The Adaptive Significance of Drought Escape in *Avena Barbata*, an Annual Grass. Evolution 60, 2478–2489. 10.1111/j.0014-3820.2006.tb01883.x 17263110

[B45] Shim J. S. Kubota A. Imaizumi T. (2017). Circadian Clock and Photoperiodic Flowering in Arabidopsis: CONSTANS Is a Hub for Signal Integration. Plant Physiol. 173, 5–15. 10.1104/pp.16.01327 27688622 PMC5210731

[B46] Simon N. M. L. Graham C. A. Comben N. E. Hetherington A. M. Dodd A. N. (2020). The Circadian Clock Influences the Long-Term Water Use Efficiency of Arabidopsis. Plant Physiol. 183, 317–330. 10.1104/pp.20.00030 32179629 PMC7210627

[B47] Soga K. Wakabayashi K. Hoson T. Kamisaka S. (1999). Inhibition of Reproductive Growth of Arabidopsis in Airtight Vessels. Adv. Space Res. 23, 2037–2040. 10.1016/s0273-1177(99)00346-4 11710388

[B48] Soga K. Wakabayashi K. Kamisaka S. Hoson T. (2002). Stimulation of Elongation Growth and Xyloglucan Breakdown in Arabidopsis Hypocotyls under Microgravity Conditions in Space. Planta 215, 1040–1046. 10.1007/s00425-002-0838-x 12355165

[B49] Strickland D. T. Campbell W. F. Salisbury F. Bingham G. E. (1997). Morphological Assessment of Reproductive Structures of Wheat Grown on Mir. Grav. Space Biol. Bull. 11, 14.

[B50] Sugimoto M. Oono Y. Gusev O. Matsumoto T. Yazawa T. Levinskikh M. A. (2014). Genome-wide Expression Analysis of Reactive Oxygen Species Gene Network in Mizuna Plants Grown in Long-Term Spaceflight. BMC Plant Biol. 14, 4. 10.1186/1471-2229-14-4 24393219 PMC3927260

[B51] Sun H. Wang J. Gong Z. Yao J. Wang Y. Xu J. (2018). Quantitative Integration of Epigenomic Variation and Transcription Factor Binding Using MAmotif Toolkit Identifies an Important Role of IRF2 as Transcription Activator at Gene Promoters. Cell Discov 4, 38. 10.1038/s41421-018-0045-y 30002873 PMC6037672

[B52] Sychev V. N. Levnskikh M. A. Gostimsky S. A. Bingham G. E. Podolsky I. G. (2003). Spaceflight Effects on Consecutive Generations of Peas Grown Onboard the Russian Segment of the International Space Station. Acta Astron. 60, 426–432. 10.1016/j.actaastro.2006.09.009

[B53] Tripathy B. C. Brown C. S. Levine H. G. Krikorian A. D. (1996). Growth and Photosynthetic Responses of Wheat Plants Grown in Space. Plant Physiol. 110, 801–806. 10.1104/pp.110.3.801 8819868 PMC157779

[B54] Ueda J. Miyamoto K. Yuda T. Hoshino T. Sato K. Fujii S. (2000). STS-95 Space experiment for Plant Growth and Development, and Auxin Polar Transport. Biol. Sci. Space 14, 47–57. 10.2187/bss.14.47 11543421

[B55] Valbuena M. A. Manzano A. Vandenbrink J. P. Pereda-Loth V. Carnero-Diaz E. Edelmann R. E. (2018). The Combined Effects of Real or Simulated Microgravity and Red-Light Photoactivation on Plant Root Meristematic Cells. Planta 248, 691–704. 10.1007/s00425-018-2930-x 29948124

[B56] Vandenbrink J. P. Herranz R. Poehlman W. L. Alex Feltus F. Villacampa A. Ciska M. (2019). RNA -seq Analyses of *Arabidopsis thaliana* Seedlings after Exposure to Blue-Light Phototropic Stimuli in Microgravity. Am. J. Bot. 106, 1466–1476. 10.1002/ajb2.1384 31709515

[B57] Wang L. Han F. Zheng H. (2018). Photoperiod-controlling Guttation and Growth of rice Seedlings under Microgravity on Board Chinese Spacelab TG-2. Microgravity Sci. Tech. 30, 834–847. 10.1007/s12217-018-9644-3

[B58] Warner J. R. (1999). The Economics of Ribosome Biosynthesis in Yeast. Trends Biochem. Sci. 24, 437–440. 10.1016/s0968-0004(99)01460-7 10542411

[B59] Wehmeyer N. Hernandez L. D. Finkelstein R. R. Vierling E. (1996). Synthesis of Small Heat-Shock Proteins Is Part of the Developmental Program of Late Seed Maturation. Plant Physiol. 112, 747–757. 10.1104/pp.112.2.747 8883386 PMC157999

[B60] Wu Y. Xie J. Wang L. Zheng H. (2020). Circumnutation and Growth of Inflorescence Stems of *Arabidopsis thaliana* in Response to Microgravity under Different Photoperiod Conditions. Life 10, 26. 10.3390/life10030026 PMC7151594 32197304

[B61] Xie J. Zheng H. (2020). Arabidopsis Flowering Induced by Photoperiod under 3-D Clinostat Rotational Simulated Microgravity. Acta Astronautica 166, 567–572. 10.1016/j.actaastro.2018.11.014

[B62] Yakir E. Hilman D. Hassidim M. Green R. M. (2007). CIRCADIAN CLOCK ASSOCIATED1 Transcript Stability and the Entrainment of the Circadian Clock in Arabidopsis. Plant Physiol. 145, 925–932. 10.1104/pp.107.103812 17873091 PMC2048808

[B63] Yin X. Wang X. Komatsu S. (2018). Phosphoproteomics: Protein Phosphorylation in Regulation of Seed Germination and Plant Growth. Curr. Protein Pept. Sci. 19, 401–412. 10.2174/1389203718666170209151048 28190389

[B64] Zhang Y. Wang L. Xie J. Zheng H. (2015). Differential Protein Expression Profiling of *Arabidopsis thaliana* Callus under Microgravity on Board the Chinese SZ-8 Spacecraft. Planta 241, 475–488. 10.1007/s00425-014-2196-x 25374148

[B65] Zhang Y. Zheng H. Q. (2015). Changes in Plastid and Mitochondria Protein Expression in Arabidopsis Thaliana Callus on Board Chinese Spacecraft SZ-8. Microgravity Sci. Technol. 27, 387–401. 10.1007/s12217-015-9431-3

[B66] Zheng H.-Q. Wei N. Chen A.-D. Wang L.-F. Zheng W.-B. Zhang T. (2008). Live Imaging Technique for Studies of Growth and Development of Chinese Cabbage under Microgravity in a Recoverable Satellite (SJ-8). Microgravity Sci. Technol. 20, 137–143. 10.1007/s12217-008-9005-8

[B67] Zheng H. Q. (2018). Flowering in Space. Microgravity Sci. Technol. 30, 783–791. 10.1007/s12217-018-9626-5

