# Supplementary material for: Transcriptomic Analysis of the Interaction Between FLOWERING LOCUS T Induction and Photoperiodic Signaling in Response to Spaceflight
Source: Front Cell Dev Biol. 2022 Feb 1;9:813246. doi: 10.3389/fcell.2021.813246 (PMC8844200; doi:10.3389/fcell.2021.813246)
Supplement: Supplementary file 5 [file DataSheet1.pdf]

Supplementary Figure S1

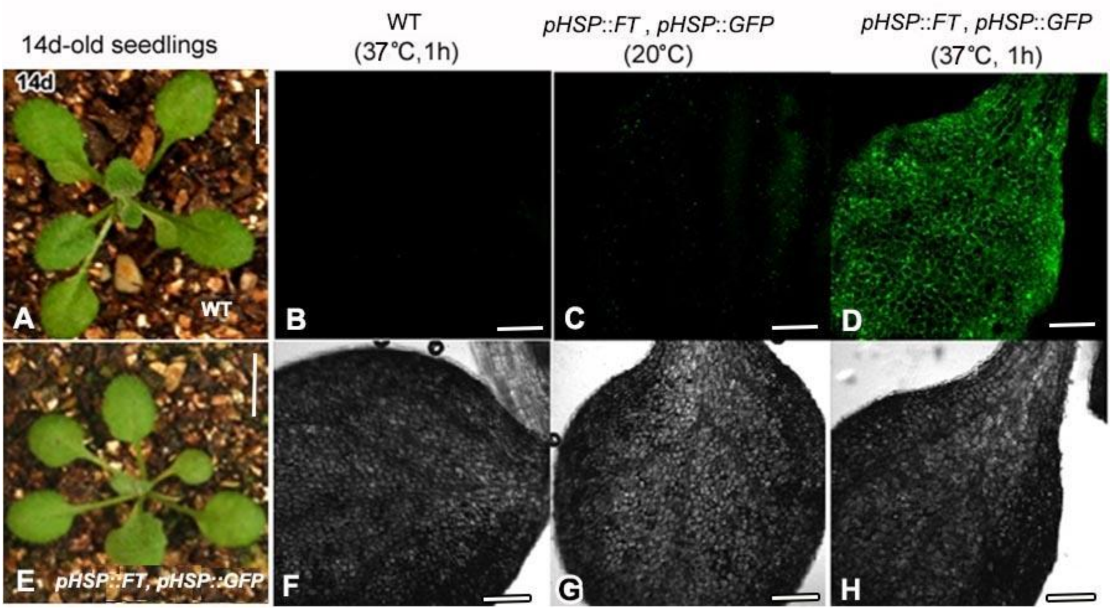

Supplemantary Figure S1 Heat shock activation of *GFP* gene expression in *pHSP::FG, pHSP*::*GFP* (FG) transigenic plants.

(A and E) A represent 14-day-old wild-type (WT) and a FG transgenic plant. Bars=10 mm.

(B-D) Fluorescence images of a leaf of a WT seedling (B) and a FG seedlings (C) treated by 37°C heating for 1h in comparison with that of the FG seedling grown under 20°C condition (C). Bars= 2 mm.

(F-H) Images of leaves corresponding to B, C and D under differential interence contrast optics microscope. Bars = 2 mm.

Supplementary Figure S2

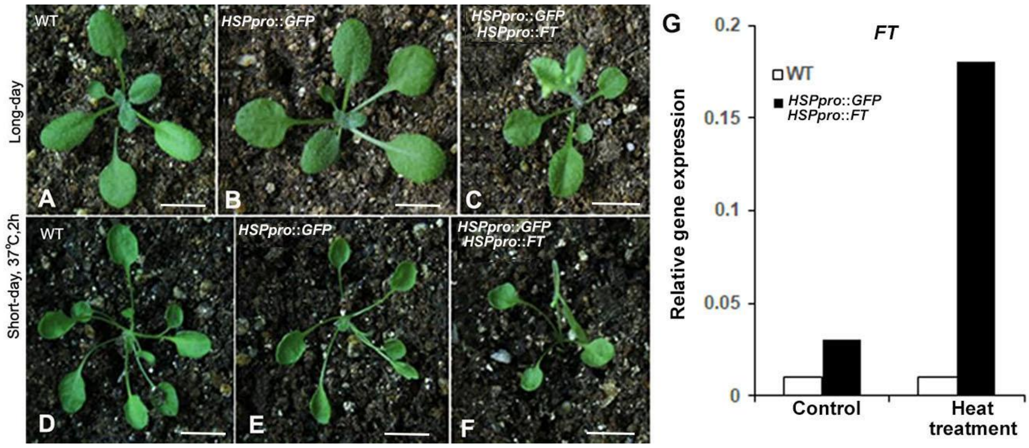

Supplementary Figure S2 Heat shock treatment induced *FT* expression and flowering. All plants were 20-day old and were subjected to heat shock (37℃) induction for 2h at day 15 after germination.

(A, D) Phonetype of wild-type (WT) plants were grown under the long-day (16 h light/8h dark, LD) and short-day (8 h light/16h dark, SD) conditions, respectively .

(B, E)Phonetype of transgenic plants *pHSP*::*GFP* were grown under the LD and the SD conditions, respectively.

(C, F) Phonetype of transgenic plants *pHSP*::*FT,pHSP::GFP* (FG) were grown under the LD and the SD conditions, respectively. Note that the FG plants under both LD- and SD-conditions (C and F) appeared early flowering in comparison with their controls of WT(A and D) and *pHSP::GFP* (B and E) under the same conditions. Bars=10 mm.

(G) qRT-PCR analysis of *FT* transcript levels in *FG* plants at day 16 grown under the SD conditions with or without the 2h heat shock treatment (37°C) at day 15 after germination. Values represent three biological replicates.

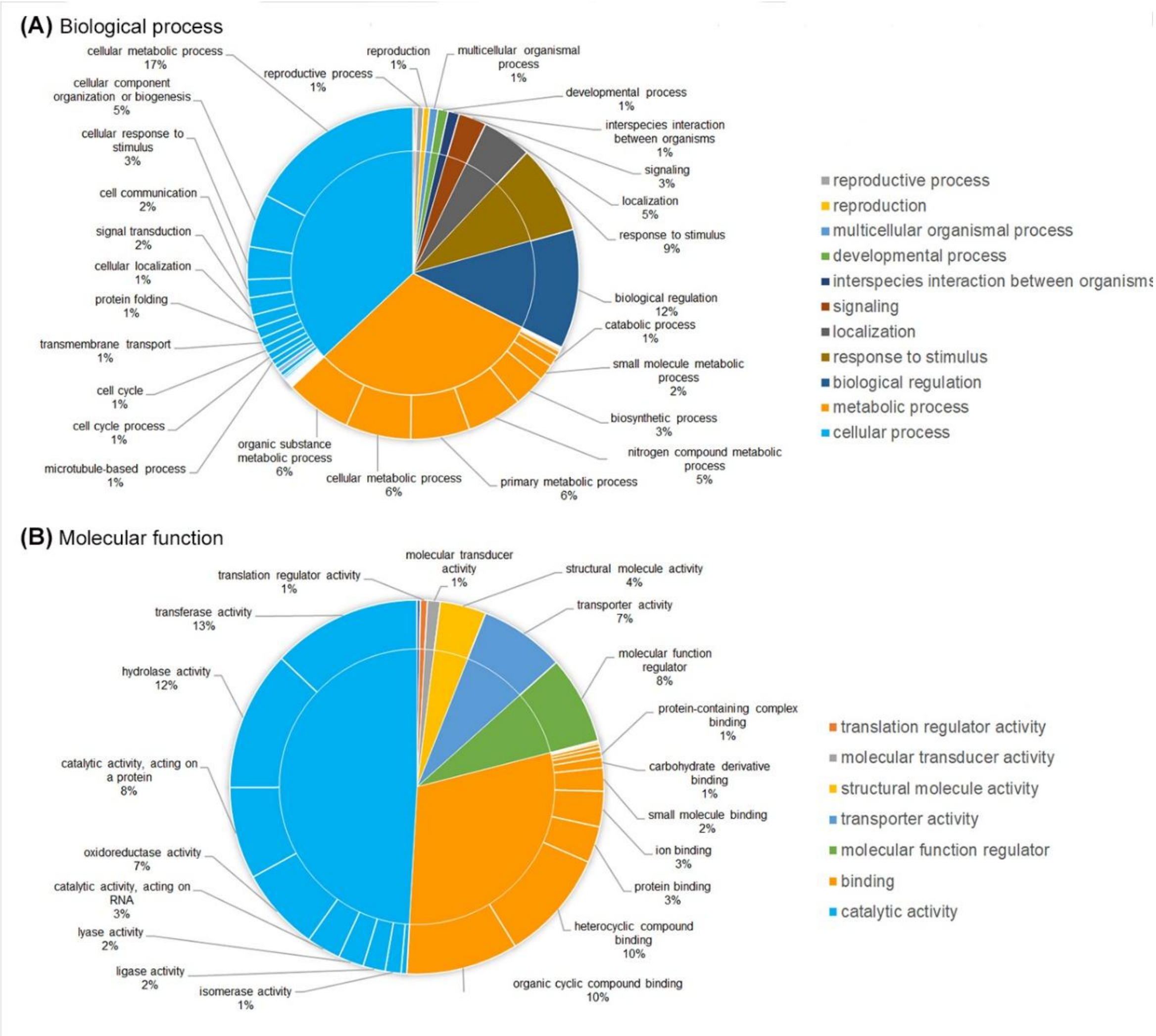

Supplementary Figure S3 PANTHER biological process (A) and molecular function (B) of differentially expressed genes. Classification of the biological systems and the functions of proteins that identified from differentially expressed genes belong to FG vs WT under the SD in space (corresponding to data Fig. 8C).
